# Coexistence of All-Order Topological States in a Three-Dimensional Phononic Topological Crystalline Insulator

**DOI:** 10.34133/research.0235

**Published:** 2023-09-13

**Authors:** Hua-Shan Lai, Hao Chen, Chu-Hao Xia, Si-Yuan Yu, Cheng He, Yan-Feng Chen

**Affiliations:** ^1^ National Laboratory of Solid State Microstructures & Department of Materials Science and Engineering, Nanjing University, Nanjing 210093, China.; ^2^ Collaborative Innovation Center of Advanced Microstructures, Nanjing University, Nanjing 210093, China.; ^3^ Jiangsu Key Laboratory of Artificial Functional Materials, Nanjing University, Nanjing 210093, China.

## Abstract

Classical-wave topological materials lacking intrinsic half-integer spins are less robust while more tunable. Here, we explore a single 3-dimensional phononic topological crystalline insulator that simultaneously exhibits a whole family of first-order quadratic surface, second-order hinge, and third-order corner states within the same bandgap. Such a topological crystalline insulator hosting all-order phases originates from the different topological nature when hierarchically projected onto different facets and lower dimensions, thus free from trivial cladding crystals. Our work offers an ideal platform for either robust wave propagation or localization in on-demand dimensions and may facilitate dimension division multiplexing technology.

## Introduction

The revelation of topological phases of matter with conserved properties under continuous deformations has created new opportunities to manipulate the quantized global behavior of solid-state materials in the past decades. To date, numerous topological phases have been recognized, including the family of quantum Hall materials [1], topological semimetals (TSMs) [2], and topological insulators (TIs) [3]. Furthermore, similar phases with different characters can hold subclasses, e.g., Dirac/Weyl/nodal-line TSMs [2], or strong/weak/crystalline TIs [4,5]. In general, their hallmarks feature robust lower-dimensional boundary states dependent on bulk topology [6]. Reducing more than one dimension is dubbed the higher-order phase [7]. Thus, 3-dimensional (3D) topological materials may hierarchically possess first-order 2D surface, second-order 1D hinge, or third-order 0D corner states. The typical cases are higher-order hinge arcs in hybrid TSMs [8] and corner states in multipole TIs [9].

On the other hand, each topological phase should relate to a particular bulk structure with specific symmetry, which seems difficult to be compatible with another one. However, by assigning multiple symmetry-protected topological index to the same bulk structure, recently proposed dual TIs can host coexisting weak TI and topological crystalline insulator (TCI) surface states on different facets of one material, such as Bi_1_Te_1_, Pt_2_HgSe_3_, and Bi_2_TeI [10–12]. Although those weak and TCI phases relying on an additionally specific discrete translation or crystalline symmetry are less robust than strong ones [4,5], they are convenient to fabricate and control. Such dual topology essentially enriches quantum manipulation of topological states, inspiring to investigate the possibility of triple topology and even associated with higher-order phases or maybe in artificially classical-wave systems [13–18].

For high-efficiency manipulation of waves, intensive effects have been devoted to realizing many classical-wave topological states over the past decade [19–26], especially in high-dimensional, non-Hermitian, and non-Abelian systems [27,28]. Particularly for classical-wave TIs, pseudospins are compromisingly constructed via artificial time-reversal, duality, and/or lattice symmetries [29]. Consequently, they are fragile [30], requiring specific symmetry trivial claddings to maintain robustness, limiting further miniaturization and application. However, their designable structure, scalable geometry, and accessible measurement advance on-demand tunable behaviors, offering the opportunity to combine various phases, such as hybrid edge and corner states within different bandgaps of a bilayer 2D model [31]. However, the coexistence of more types of topological phases in 3D is still challenging, especially within the same frequency window.

**Fig. 1.**
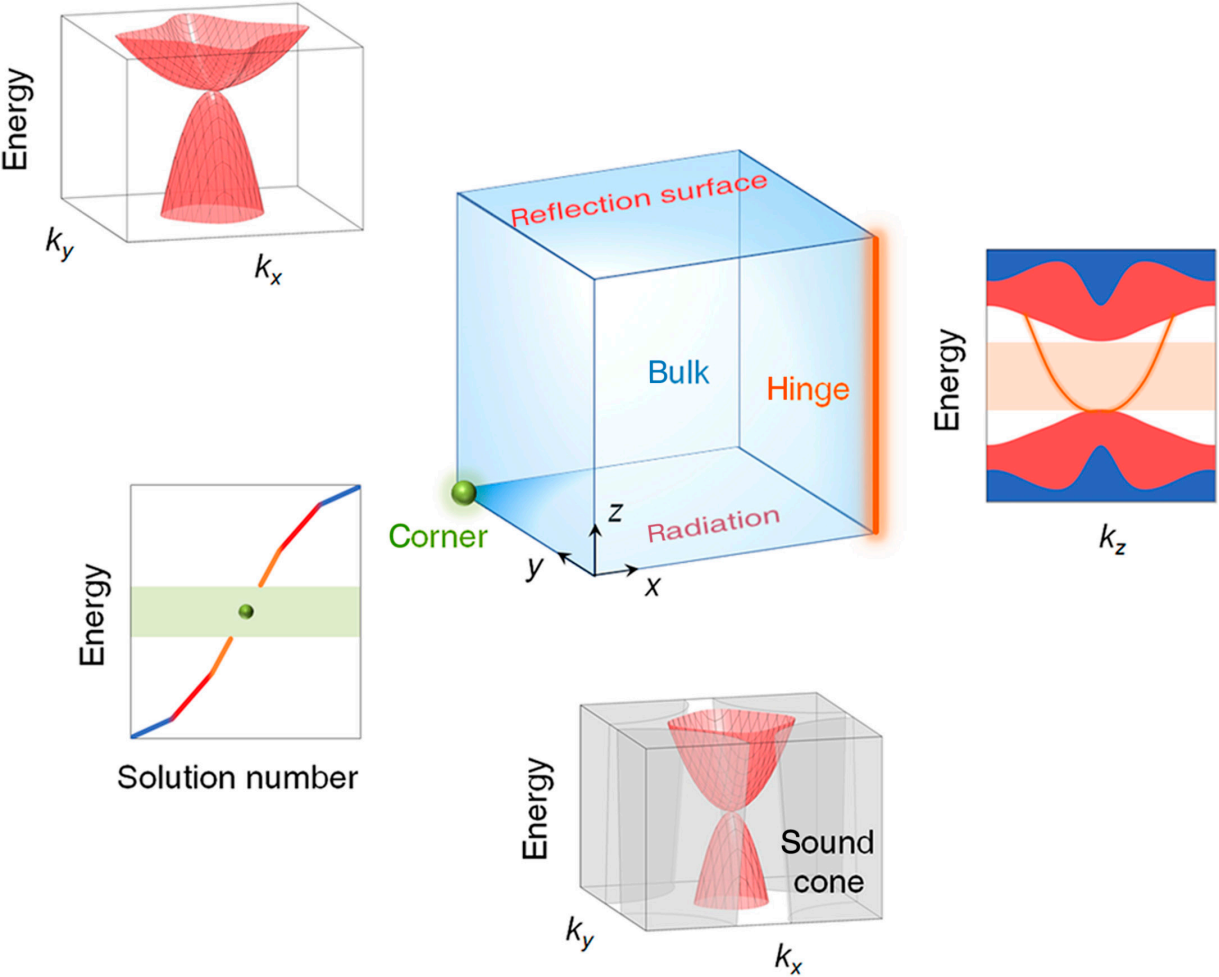
Schematics of coexistence of multiple overlapping 2D surface, 1D *k_z_* hinge, and 0D corner states in one 3D phononic structure. The color blue, red, orange, and green represent bulk, surface, hinge, and corner states, respectively. The unique quadratic surface states as hallmarks feature of TCI are supported on both top (reflection) and bottom (radiation) facets. In addition, the lateral facet can open up a bandgap to support the higher-order hinge state. The corner state can also be realized by tailoring lateral facets to further gap hinge states.

In this work, we explore a 3D phononic TCI exhibiting both different and hierarchical topological natures, which simultaneously hosts all-order topological phases within the same frequency window. As shown in Fig. 1, the top facet with reflection boundary supports quadratic 2D surface states acting as a phononic TCI. At the same time, the appearing 1D *k_z_* hinge state originates from the fractional hinge charge, while the 0D corner state inherits from the higher-order topology of the lateral facet. We observe the first self-guiding topological surface states for sound on a radiation bottom facet. This coexistence of all lower-dimensional boundary states provides a versatile platform for separately yet robustly manipulating sound in on-demand dimensions of one material.

## Results

### Bulk topological property

**Fig. 2.**
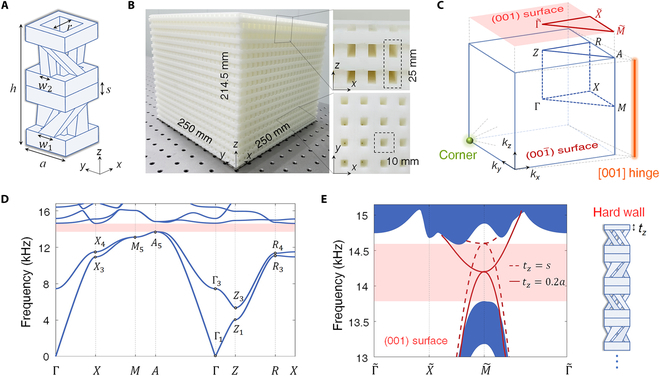
Bulk and surface states dispersion of the phononic crystal. (A) A unit cell of bilayer tetragonal lattice with opposite chirality of spiral waveguides, wherein sound propagates. The parameters *a* = 10 mm, *h* = 25 mm, *r* = 6 mm, *w*_1_ = 4 mm, *w*_2_ = 2.5 mm, and *s* = 3.25 mm. (B) Sample photograph with magnified lateral and bottom views. (C) 3D bulk BZ and its projections to lower dimensions. (D) Calculated bulk band structures marked with irreducible representations (irreps) of the little group at high-symmetry points. The labels of all irreps in space group *P*422 can be obtained via the Bilbao Crystallographic Server [38]. (E) Projected band structures on reflection (001) facet. The dashed (solid) red lines represent surface states without (with) truncation *t_z_* = *s* (0.2*a*), as a supercell configuration shown in the right panel.

Here, our 3D phononic crystal comprises stacked bilayer square-loop air cavities connecting with 4 uniform spiral waveguides in each layer but with different chirality in adjacent layers, fabricated via 3D printing with opposite geometries against air cavities (Fig. 2A to C). In a theoretical tight-binding model, each layer has 2 effective atoms (resonant air cavities), while spiral waveguides with radius *w*_1_/*w*_2_ control the interlayer coupling strength. For the opposite chirality but identical size case (*w*_1_ = *w*_2_), a 4-fold degenerated node protected by the *z*-axis mirror symmetry of *D*_4*h*_ will appear at *A* point in Brillouin zone (BZ) (Section S2). Detuning the interlayer couplings to break such mirror, e.g., *w*_1_ > *w*_2_ (the topologically trivial case *w*_1_ < *w*_2_ is discussed in Section S3), we lift the degeneracy to obtain a complete bandgap above the first 2 bands, as shown in Fig. 2D. This lattice belongs to *P*422 (no. 89 space group) with *C*_4*z*_ and *C*_2*x/y*_ rotation symmetries. Then, 2-fold degenerated Bloch states at *A* point can hybridize into artificial spins for acoustic waves.

### Symmetry-forced quadratic surface states

On (001) facet with reflection boundary (hard wall), a pair of gapless surface states with a quadratic node appears in the bandgap, protected by *C*_4*z*_ rotation and time-reversal symmetry, acting as a phononic analog of Fu’s model [5]. The gapless behavior remains unchanged under moderately truncating the surface, only with a shift of the surface node (Fig. 2E). To characterize the topology, we tracked the evolution of Berry phase of the lower 2 bands through Wilson loop along the *k_z_* direction. The spectral flow of Berry phase that winds the whole [−π π] range is a notable sign of 3D TCI featured with gapless (001) surface states (Section S3). The quadratic surface states help investigate exotic phenomena such as extremal transmission (Section S9) and tunneling [15]. Further breaking *C*_4*z*_ symmetry to *C*_2*z*_, quadratic surface states will split into 2 linear surface Dirac cones with various tilted fashions to broaden the phase category of our system (Section S5).

**Fig. 3.**
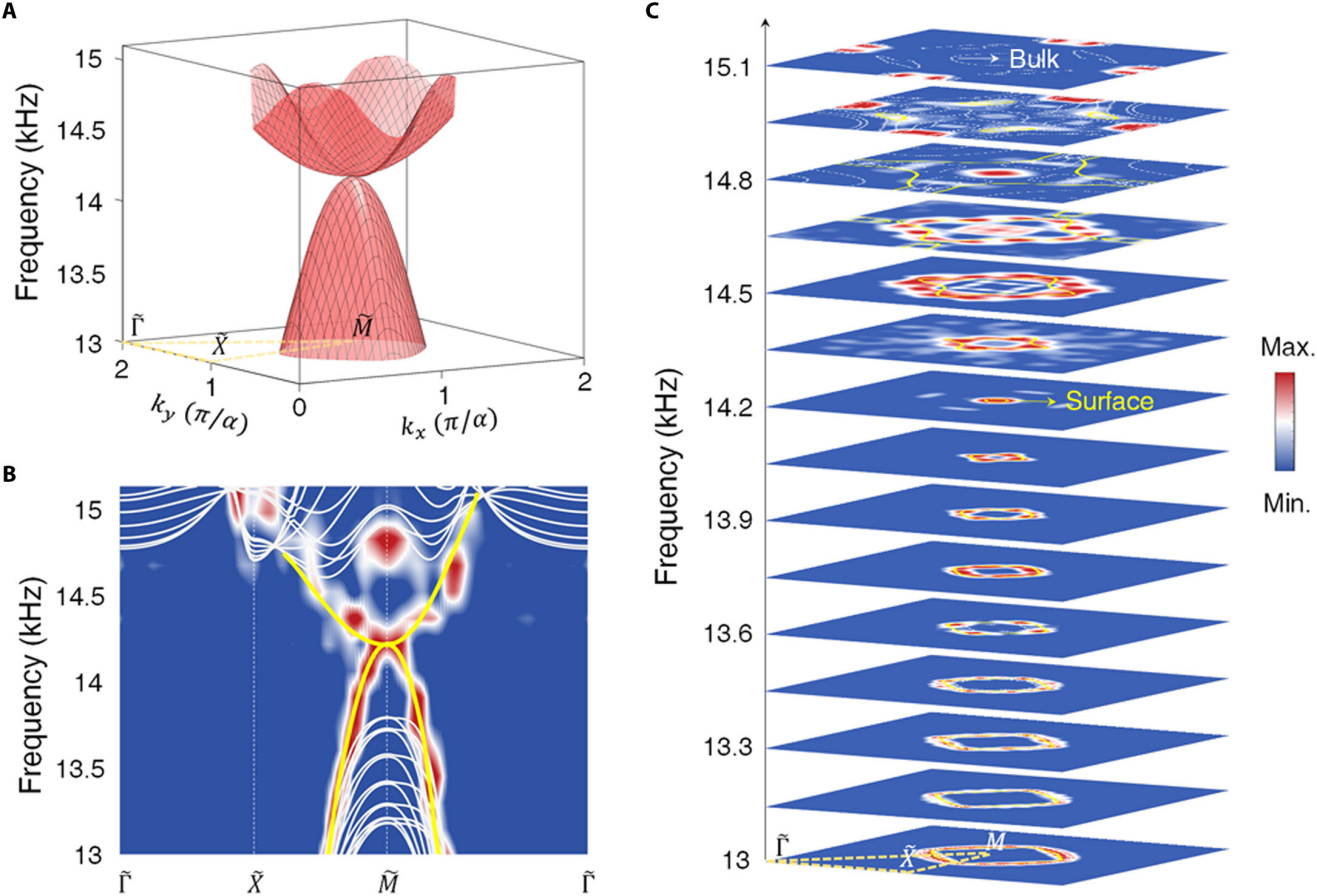
Observation of quadratic surface states for sound on a reflection facet. (A) 3D view of calculated quadratic surface states. (B) Measured surface dispersion (color scale) along high-symmetry lines of surface BZ. (C) Measured isofrequency surface arcs (color scale). The yellow (white) lines denote numerical surface (bulk) states.

In experiments, we tailor the (001) facet with *t_z_* = 0.2*a* to ensure the surface node locates in the middle of the bulk bandgap. A 3D view of numerical surface states (Fig. 3A) shows the quadratic dispersion. A 2-mm-thick resin plate is covered onto the (001) facet as a reflection boundary. Via Fourier transforming the experimentally measured surface acoustic pressure fields, we map out the surface dispersion along high-symmetry lines of surface BZ, matching well with theoretical results (Fig. 3B). To further identify the quadratic fashion of surface states, we present measured isofrequency surface arcs in a wide frequency window from 13 to 15.1 kHz with an increment of 0.15 kHz (Fig. 3C). As expected, the surface node appears approximately at 14.2 kHz.

However, the $\left(00\bar{1}\right)$ facet is trivial with a reflection boundary due to the chiral structure. This 3D phononic TCI, whose surface states are not solely dependent on the bulk topology, is a fragile phase without rigorous bulk-boundary correspondence [30]. Here, we resort to elementary band representation analysis [32]. According to symmetry data vectors of high-symmetry points in bulk BZ, we get the decomposition of the lower 2 bands (Section S3):

$${\left(A_{1}\right)}_{2e}⊕{\left(A_{2}\right)}_{1c}⊖{\left(B_{1}\right)}_{1a},$$ (1)

where ⊖ denotes the missing elementary band representation induced from Wyckoff position 1*a*, demonstrating the fragile phase. Compared to the electronic TCIs, the fermionic-like artificial spins in our phononic also rely on crystalline symmetries, making it less stable. Nevertheless, the surface states are still robust against moderate perturbations and possess proper topological protection (Section S10). At the same time, such fragile characters could bring favorite tunable behavior, which is vital for classical waves.

### Self-guiding surface states for sound

On the other hand, unlike naturally bounded electrons, the topological states for airborne sound usually suffer from radiation leakage. The self-guiding topological states without any ancillary confinement, which allow waves access to the free space, are preferred [18]. In our case, the $\left(00\bar{1}\right)$ facet can support self-guiding topological surface states adjacent to air. The difference between top and bottom facets comes from their different coupling strengths. Attributed to the fragile character, for the $\left(00\bar{1}\right)$ facet, in-gap quadratic surface states can be entirely removed under a reflection boundary but kept intact under a radiation boundary (Section S4). From the polarization view, the reflection or radiation boundary possessing different surface onsite potentials is compatible only with a specific mode [14].

**Fig. 4.**
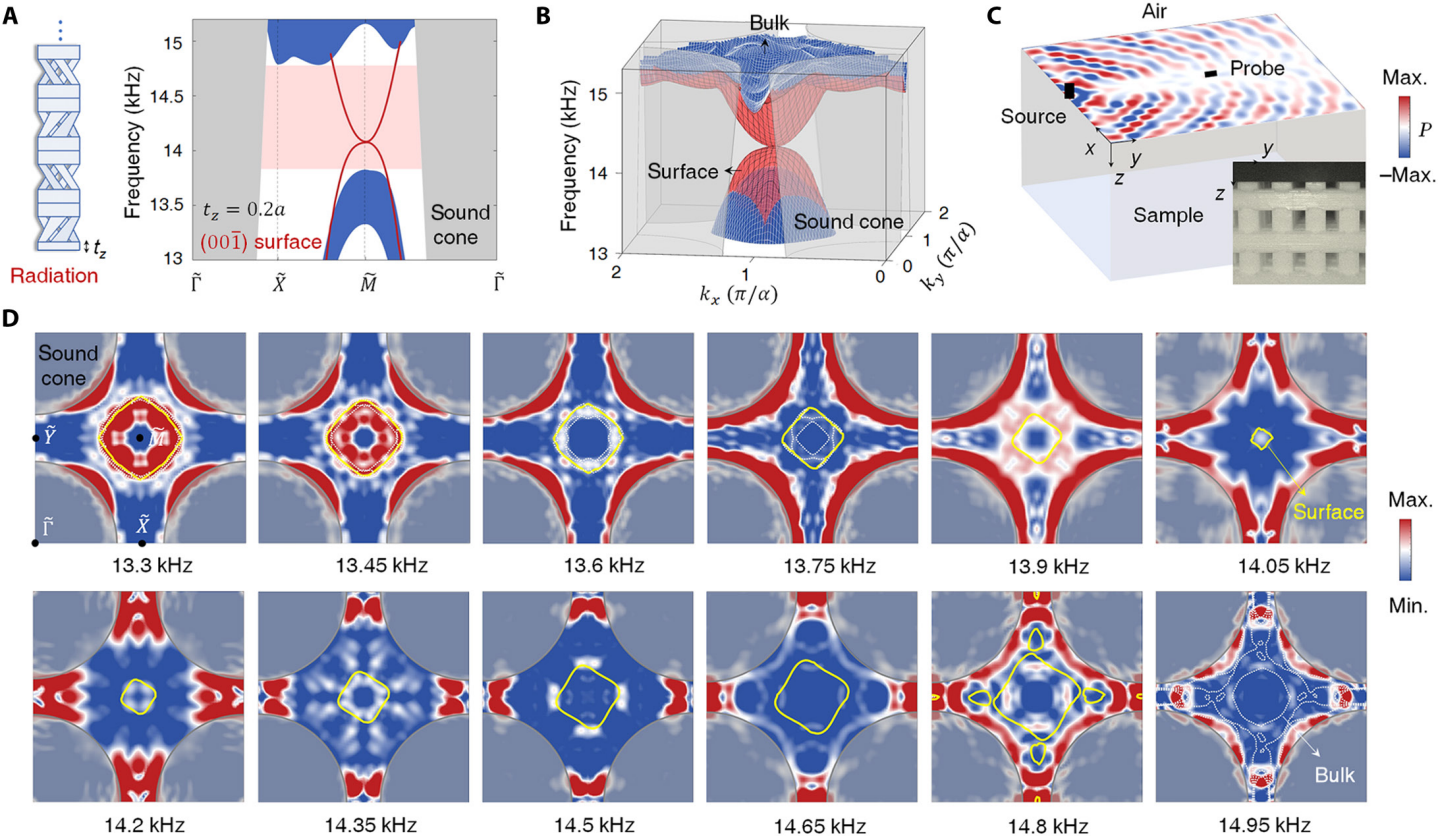
Observation of self-guiding surface states for sound on a radiation facet. (A) Supercell configuration and projected band structures on $\left(00\bar{1}\right)$ facet with radiation boundary. (B) 3D view of calculated surface states. (C) Schematic of the experimental setup with measured surface acoustic pressure at 13.45 kHz. The inset shows the truncated facet (*t_z_* = 0.2*a*). (D) Experimentally measured isofrequency contours (color scale). The yellow (white) lines and gray shadow regions denote numerical surface (bulk) states and sound cone, respectively.

As shown in Fig. 4A and B, numerical surface states on the $\left(00\bar{1}\right)$ facet locate totally outside the sound cone, perfectly supporting self-guiding surface waves. A point-like source is placed to excite surface modes (Fig. 4C). The measured isofrequency contours in Fig. 4D are in good agreement with theoretical results. The surface node is approximately at 14.05 kHz in this case, slightly lower than that in the reflection (001) facet case. The inevitable signals for the air modes owe to the impedance mismatching between the excitation source and large wave vectors of self-guiding surface modes.

### Higher-order hinge and corner states

**Fig. 5.**
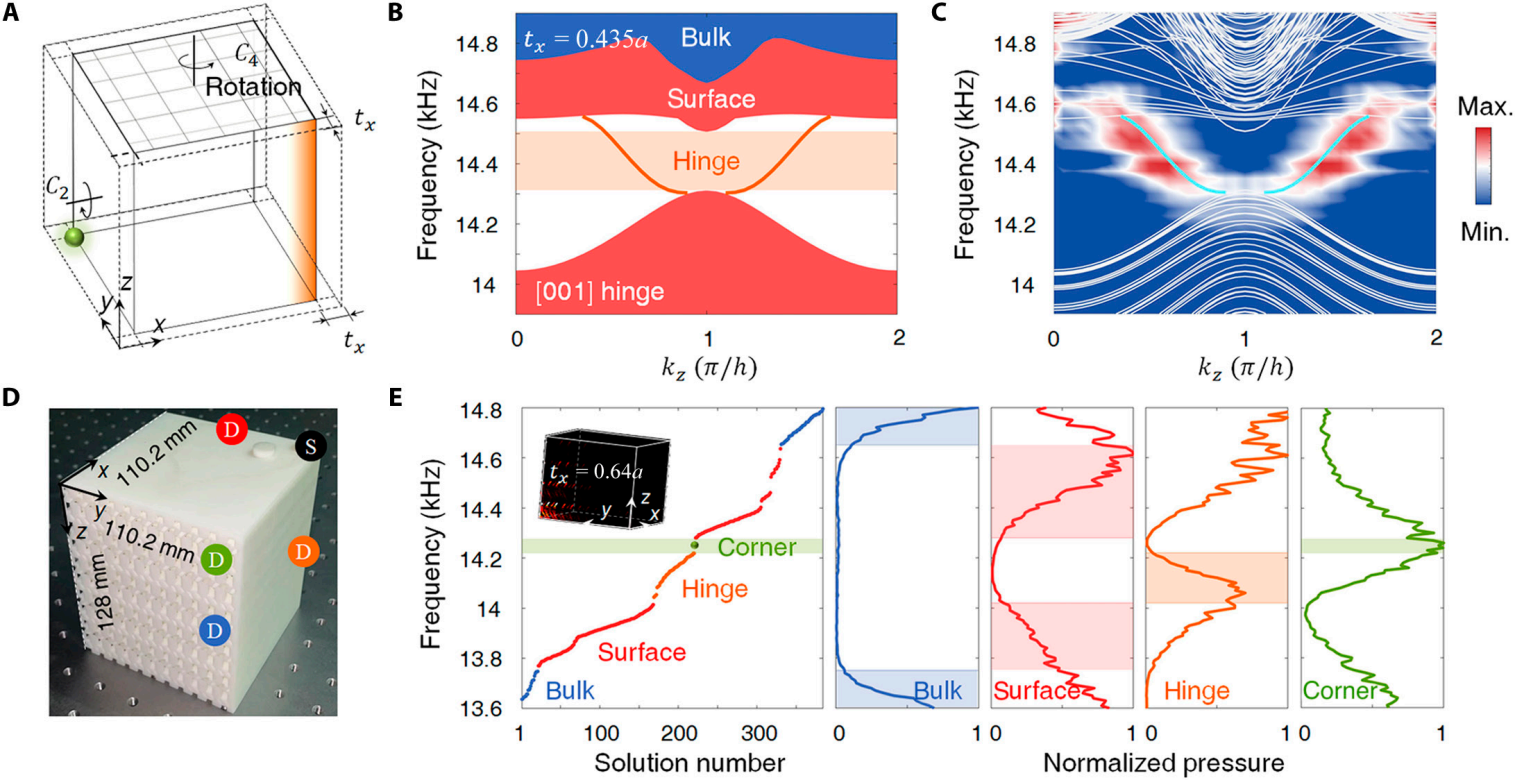
Higher-order hinge and corner states. (A) Schematic of truncated lateral facets. (B) Calculated [001] hinge states with *t_x_* = 0.435*a*. (C) Measured hinge spectrum (color scale), where the cyan line represents the numerical hinge dispersion. (D) A photograph of a small-size sample (*t_x_* = 0.64*a*) to measure the corner state, where the positions for the source (dark) and detectors for the bulk (blue), surface (red), hinge (orange), and corner (green) are marked. (E) Calculated eigen frequencies and measured bulk, surface, hinge, and corner response spectra. The inset shows simulated acoustic field distribution (amplitude) of the corner state.

Besides first-order surface states, our 3D phononic TCI also hosts second-order hinge states coexisting within the same bandgap. In contrast to the top or bottom facets with *C*_4*z*_ symmetry, the lateral facet with *C*_2*x/y*_ rotation cannot satisfy degeneracy to spontaneously support gapless topological surface states. However, its anisotropic behavior enables the existence of a higher-order [001] hinge state where 2 gapped lateral facets join, independent of the TCI case. Here, we tailor lateral facets with *t_x_* = 0.435*a* (Fig. 5A) to obtain a complete surface bandgap from 14.3 to 14.5 kHz, in which the [001] hinge state appears (Fig. 5B), agreeing with our experimentally measured hinge dispersion (Fig. 5C).

This nontrivial [001] hinge state originates from the fractional quantization of hinge charge due to the filling anomaly. We map the 3D system into a series of *k_z_*-dependent 2D slices after Fourier transformation in the dimensional reduction procedure. Then, the energetically gapped 2D insulator with *C*_4*z*_ symmetry can be classified into distinct phases in terms of rotation topological invariants [33]:

$$\chi^{4}=\left(\left[X_{1}^{\left(2\right)}\right],\left[M_{1}^{\left(4\right)}\right],\left[M_{2}^{\left(4\right)}\right]\right).$$ (2)

By analyzing the C_4*z*_-related properties of Bloch wave functions on high-symmetry points, we get the nontrivial topological index *χ*^4^ = (−2, −2, 1) for each *k_z_* slice, inducing localization modes at the corner of a finite sample. Therefore, the stacked 3D geometry hosts the second-order [001] hinge state with fractional hinge charge, located within the lateral surface bandgap.

Next, we tailor the [001] hinge (*t_x_* = 0.64*a*) to open a complete bandgap, making it feasible to host the third-order corner state [34]. In this case, we gap $\left(00\bar{1}\right)$ surface states, including [100] and [010] hinges as well. In experiments, we fabricate a small-size sample with the same bulk structure (Fig. 5D), where the corner of interest is the intersecting point of 3 facets covered by hard walls. As shown in Fig. 5E, the measured response spectra for the bulk, surface, hinge, and corner states coincide with theoretical results. Because of the loss and shallow hinge bandgap, the response frequency of the corner state will overlap with those of hinges, resulting in a broad response spectrum with a relatively low-quality factor (Section S11). This corner state is attributed to the higher-order topology of the *C*_2*y*_-invariant (010) facet [or *C*_2*x*_-invariant (100) facet] under elaborately designed boundary truncation. The topology can be characterized by surface rotation invariants [33]:

$$\chi^{2}=\left(\left[{\bar{Z}}_{1}^{\left(2\right)}\right],\left[{\bar{Y}}_{1}^{\left(2\right)}\right],\left[{\bar{A}}_{1}^{\left(2\right)}\right]\right),$$ (3)

which gives the nontrivial topological index *χ*^2^ = (0, 0, −1) to support the corner state (Section S8).

## Discussion

Despite the lack of rigorous bulk-boundary correspondence and strict topological protection in fragile phases, these multiple boundary states, once in existence, are still robust against various moderate disorders and defects, fundamentally distinct from trivial ones (Section S10). Notably, our design holds potential to exhibit more other topological phases. For example, the lateral facets can act as phononic analogs of quantum relativistic Jackiw–Rebbi states [35] with opposite effective masses [16]. In addition, the [100] or [010] hinge could also be topological, even self-guiding, either for hierarchical cases related to surface degenerated nodes or for valley-induced cases within a complete bandgap (Section S7). Moreover, our model will become a hybrid semimetal case for the identical interlayer couplings [36]. In addition, the quadrupole hinge or octupole corner states can be expected by introducing negative couplings [9].

To conclude, by fully using the designable advantage of 3D materials, we unveil the feasibility of harnessing all-order topological phases in a single sample, other than previous different structures [16,37]. In principle, all 6 2D faces, 12 1D edges, and 8 0D vertices of such a cuboid can simultaneously support topological states. Different from the coexisting case based on the same or similar bulk polarizations [34], our all-order topological states originate from different topological natures projected on different dimensions. Thus, they can overlap at the same frequency, enabling separate manipulation in on-demand dimensions or interaction in cross-dimensions of one material. Furthermore, the multiple overlapping lower-dimensional boundary states severing as individual signal processing modules may facilitate a unique type of spatial dimension division multiplexing technique for topological communication and antenna arrays.

## Materials and Methods

### Experimental design

Our experiments are performed for airborne sounds in an audible frequency window from 13 to 15.1 kHz. In the experiment, a commercial loudspeaker (AMT-47) with a horn acts as a point-like source. The homemade microphone has a full dimension of 5 mm × 2 mm × 3.5 mm, small enough to insert into the sample to obtain acoustic pressure. Compared to a commercial 1/4-inch microphone (BSWA-MPA416 from BSWA Technology), our homemade microphone shows a similar flat response (<5 dB) in the frequency window of 11 to 16 kHz (more details in the Supplementary Materials).

Considering the aspect of experimental validity and economical principles, we divide into 3 small segments from a large crystal to measure the different dimensional boundary states. Compared to a large-size sample with a full dimension of 25*a* × 25*a* × 24*h*, the designing strategy using different samples of different sizes (25*a* × 25*a* × 8*h* for surface, 6*a* × 6*a* × 24*h* for hinge, and 11*a* × 11*a* × 5*h* for corner) can effectively save about half of the raw materials. Such division is also convenient for excitation and measurement in experiments under various boundary conditions.

All samples used in the experiments are fabricated with photosensitive resin (Godart 8111X) via 3D printing (tolerance, 0.1 mm). This stereolithography material (modulus, 3,160 MPa; density, 1.14 g·cm^−3^) acts as a hard boundary for sound due to the huge impedance mismatch. The thickness of the cover plate severed as a hard boundary of our samples is 2 mm.

### Statistical analysis

To measure the (001) and $\left(00\bar{1}\right)$ surface states, we use a sample with 25 × 25 × 8 unit cells. A point-like source is placed at the edge center of the surface. The amplitude and phase information in surface units are analyzed with NI cDAQ-9185 (NI 9250 and NI 9260). Following a 2D Fourier transform, the real-space data turned into the band dispersion in momentum space. Because of the *C*_4_ symmetry of the *xy* surface, the experimentally measured acoustic field distributions can be rotated to obtain another 3 *C*_4_-related parts. By averaging these 4 fields, we can map out surface arcs at a fixed frequency (Figs. 3C and 4D). We also extract the frequency-dependent surface dispersion along high-symmetry lines of the surface BZ (Fig. 3B). It should be noticed that, in Fig. 4D, only the air modes near the boundary of the sound cone with *k_z_* = 0 can be well excited. Moreover, from 14.2 to 14.65 kHz, we focus on excitation of antisymmetry modes of the upper branch of surface states (usually deemed as deaf modes), which makes the air modes relatively weak and discontinuous.

To measure [001] hinge dispersion (Fig. 5C), we use the sample with 5 × 5 units along the *x* and *y* directions and 22 units along the z direction. We detect 22 points at each unit cell under frequency sweep. To measure the corner state, we use a small-size sample with the same bulk structure, having the dimensions of 110.2 mm × 110.2 mm × 128 mm. All 3 adjacent truncated faces are covered with 2-mm-thick layers. We design a hole in the corner (black marker in Fig. 5D) to place the source. The detector is inserted into the sample to obtain the bulk, surface, hinge, and corner response spectra.

### Simulations

The full-wave simulations are implemented by the commercial software COMSOL Multiphysics with the 3D Acoustic module based on the finite element method. The mass density and sound velocity are chosen to be 1.25 kg·m^−3^ and 343 m·s^−1^, respectively. The bulk band structures (Fig. 2D) are calculated for the unit cell (Fig. 2A) with periodic Bloch boundary conditions (PBCs) in 3 orthogonal directions. In the calculation of surface states and projected band structures, a *z*-direction supercell with PBCs along the *x* and *y* axes is used. Along the *z* direction, the hall wall boundary is adopted in Fig. 2E, while the radiation boundary is adopted in Fig. 4A. In each case, the supercell is thick enough to avoid the coupling between the 2 surface states on different facets. It should be noticed that the quadratic surface states in Fig. 4A would hybridize with the air modes when an additional air layer instead of the radiation boundary is adopted on the bottom $\left(00\bar{1}\right)$ facet. This hybridization does not affect the quadratic surface node at $\tilde{M}$, but only with fluctuations of the surface dispersion near sound lines [18]. In the calculation of the hinge dispersion in Fig. 5B, a slab-liked supercell with terminations specified in the text is used. The PBCs are applied along the *z* direction, while hard wall boundaries are applied for the other 2 directions. As for the calculations of the corner spectrum in Fig. 5E, the hard boundary is adopted on all 6 facets.

## Data Availability

All data are available within the main text and the Supplementary Materials.

## Supplementary Materials

Supplementary 1 Sections S1 to S12 Figs. S1 to S22 References [39,40]

## References

[1] Klitzing KV, Dorda G, Pepper M. New method for high-accuracy determination of the fine-structure constant based on quantized Hall resistance. Phys Rev Lett. 1980;45(6):494–497.

[2] Armitage NP, Mele EJ, Vishwanath A. Weyl and Dirac semimetals in three-dimensional solids. Rev Mod Phys. 2018;90(1): Article 015001.

[3] Hasan MZ, Kane CL. Colloquium: Topological insulators. Rev Mod Phys. 2010;82(4):3045–3067.

[4] Fu L, Kane CL, Mele EJ. Topological insulators in three dimensions. Phys Rev Lett. 2007;98(10): Article 106803. 17358555 10.1103/PhysRevLett.98.106803

[5] Fu L. Topological crystalline insulators. Phys Rev Lett. 2011;106(10): Article 106802. 21469822 10.1103/PhysRevLett.106.106802

[6] Halperin BI. Quantized Hall conductance, current-carrying edge states, and the existence of extended states in a two-dimensional disordered potential. Phys Rev B. 1982;25(4):2185–2190.

[7] Benalcazar WA, Bernevig BA, Hughes TL. Quantized electric multipole insulators. Science. 2017;357(6346):61–66. 28684520 10.1126/science.aah6442

[8] Ghorashi SAA, Li T, Hughes TL. Higher-order Weyl semimetals. Phys Rev Lett. 2020;125(14): Article 266804. 33449787 10.1103/PhysRevLett.125.266804

[9] Serra-Garcia M, Peri V, Susstrunk R, Bilal OR, Larsen T, Villanueva LG, Huber SD. Observation of a phononic quadrupole topological insulator. Nature. 2018;555(7696):342–345. 29334685 10.1038/nature25156

[10] Eschbach M, Lanius M, Niu C, Mlynczak E, Gospodaric P, Kellner J, Schuffelgen P, Gehlmann M, Doring S, Neumann E, et al. Bi_1_Te_1_ is a dual topological insulator. Nat Commun. 2017;8:14976. 28429708 10.1038/ncomms14976 PMC5413958

[11] Facio JI, Das SK, Zhang Y, Koepernik K, van den Brink J, Fulga IC. Dual topology in jacutingaite Pt_2_HgSe_3_. Phys Rev Mater. 2019;3(7): Article 074202.

[12] Avraham N, Kumar Nayak A, Steinbok A, Norris A, Fu H, Sun Y, Qi Y, Pan L, Isaeva A, Zeugner A, et al. Visualizing coexisting surface states in the weak and crystalline topological insulator Bi_2_TeI. Nat Mater. 2020;19(6):610–616. 32203460 10.1038/s41563-020-0651-6

[13] Lu L, Fang C, Fu L, Johnson SG, Joannopoulos JD, Soljacic M. Symmetry-protected topological photonic crystal in three dimensions. Nat Phys. 2016;12:337–340.

[14] Ochiai T. Gapless surface states originating from accidentally degenerate quadratic band touching in a three-dimensional tetragonal photonic crystal. Phys Rev A. 2017;96(4): Article 043842.

[15] Liu Y, Xu Y, Duan W. Three-dimensional topological states of phonons with tunable pseudospin physics. Research. 2019;2019:5173580. 31549065 10.34133/2019/5173580 PMC6750063

[16] He C, Lai H-S, He B, Yu S-Y, Xu X, Lu M-H, Chen Y-F. Acoustic analogues of three-dimensional topological insulators. Nat Commun. 2020;11:2318. 32385317 10.1038/s41467-020-16131-w PMC7211004

[17] Kobayashi S, Furusaki A. Fragile topological insulators protected by rotation symmetry without spin-orbit coupling. Phys Rev B. 2021;104(19): Article 195114.

[18] Kim M, Wang Z, Yang Y, Teo HT, Rho J, Zhang B. Three-dimensional photonic topological insulator without spin–orbit coupling. Nat Commun. 2022;13:3499. 35715401 10.1038/s41467-022-30909-0 PMC9205999

[19] Ozawa T, Price HM, Amo A, Goldman N, Hafezi M, Lu L, Rechtsman MC, Schuster D, Simon J, Zilberberg O, et al. Topological photonics. Rev Mod Phys. 2019;91: Article 015006.

[20] Ma G, Xiao M, Chan CT. Topological phases in acoustic and mechanical systems. Nat Rev Phys. 2019;1:281–294.

[21] Haldane FD, Raghu S. Possible realization of directional optical waveguides in photonic crystals with broken time-reversal symmetry. Phys Rev Lett. 2008;100(1): Article 013904. 18232766 10.1103/PhysRevLett.100.013904

[22] Wang Z, Chong Y, Joannopoulos JD, Soljacic M. Observation of unidirectional backscattering-immune topological electromagnetic states. Nature. 2009;461(7265):772–775. 19812669 10.1038/nature08293

[23] Rechtsman MC, Zeuner JM, Plotnik Y, Lumer Y, Podolsky D, Dreisow F, Nolte S, Segev M, Szameit A. Photonic Floquet topological insulators. Nature. 2013;496(7444):196–200. 23579677 10.1038/nature12066

[24] He C, Ni X, Ge H, Sun X-C, Chen Y-B, Lu M-H, Liu X-P, Chen Y-F. Acoustic topological insulator and robust one-way sound transport. Nat Phys. 2016;12:1124–1129.

[25] Yan Q, Liu R, Yan Z, Liu B, Chen H, Wang Z, Lu L. Experimental discovery of nodal chains. Nat Phys. 2018;14:461–464.

[26] Lin ZK, Wu Y, Jiang B, Liu Y, Wu SQ, Li F, Jiang JH. Topological Wannier cycles induced by sub-unit-cell artificial gauge flux in a sonic crystal. Nat Mater. 2022;21:430–437. 35314775 10.1038/s41563-022-01200-w

[27] Hu B, Zhang Z, Zhang H, Zheng L, Xiong W, Yue Z, Wang X, Xu J, Cheng Y, Liu X, et al. Non-Hermitian topological whispering gallery. Nature. 2021;597(7878):655–659. 34588672 10.1038/s41586-021-03833-4

[28] Chen Z-G, Zhang R-Y, Chan CT, Ma G. Classical non-Abelian braiding of acoustic modes. Nat Phys. 2021;18:179–184.

[29] Silveirinha MG. P·T·D symmetry-protected scattering anomaly in optics. Phys Rev B. 2017;95(3): Article 035153.

[30] Alexandradinata A, Höller J, Wang C, Cheng H, Lu L. Crystallographic splitting theorem for band representations and fragile topological photonic crystals. Phys Rev B. 2020;102(11): Article 115117.

[31] Yang Y, Lu J, Yan M, Huang X, Deng W, Liu Z. Hybrid-order topological insulators in a phononic crystal. Phys Rev Lett. 2021;126(15): Article 156801. 33929222 10.1103/PhysRevLett.126.156801

[32] Peri V, Song Z-D, Serra-Garcia M, Engeler P, Queiroz R, Huang X, Deng W, Liu Z, Bernevig BA, Huber SD. Experimental characterization of fragile topology in an acoustic metamaterial. Science. 2020;367(6479):797–800. 32054764 10.1126/science.aaz7654

[33] Benalcazar WA, Li T, Hughes TL. Quantization of fractional corner charge in Cn-symmetric higher-order topological crystalline insulators. Phys Rev B. 2019;99(24): Article 245151.

[34] Weiner M, Ni X, Li M, Alù A, Khanikaev Alexander B. Demonstration of a third-order hierarchy of topological states in a three-dimensional acoustic metamaterial. Sci Adv. 2020;6(13):eaay4166. 32258398 10.1126/sciadv.aay4166 PMC7101231

[35] Jackiw R, Rebbi C. Solitons with fermion number 1/2. Phys Rev D. 1976;13(12):3398–3409.

[36] Qiu H, Xiao M, Zhang F, Qiu C. Higher-order Dirac sonic crystals. Phys Rev Lett. 2021;127(14): Article 146601. 34652168 10.1103/PhysRevLett.127.146601

[37] Zheng LY, Christensen J. Dirac hierarchy in acoustic topological insulators. Phys Rev Lett. 2021;127(15): Article 156401. 34678007 10.1103/PhysRevLett.127.156401

[38] Bradlyn B, Elcoro L, Cano J, Vergniory MG, Wang Z, Felser C, Aroyo MI, Bernevig BA. Topological quantum chemistry. Nature. 2017;547:298–305. 28726818 10.1038/nature23268

[39] Taherinejad M, Garrity KF, Vanderbilt D. Wannier center sheets in topological insulators. Phys Rev B. 2014;89(11): Article 115102.

[40] Zhang X, Liu Z. Extremal transmission and beating effect of acoustic waves in two-dimensional sonic crystals. Phys Rev Lett. 2008;101(26): Article 264303. 19437645 10.1103/PhysRevLett.101.264303

