# Supplementary material for: Coexistence of All-Order Topological States in a Three-Dimensional Phononic Topological Crystalline Insulator
Source: Research (Wash D C). 2023 Sep 13;6:0235. doi: 10.34133/research.0235 (PMC10499307; doi:10.34133/research.0235)
Supplement: Supplementary 1 — Sections S1 to S12 Figs. S1 to S22 References [39,40] [file research.0235.f1.docx]

Supplementary Materials

**Coexistence of all-order topological states in a three-dimensional phononic topological crystalline insulator**

Hua-Shan Lai^1^, Hao Chen^1^, Chu-Hao Xia^1^, Si-Yuan Yu^1,2,3^, Cheng He^1,2,3,*^, Yan-Feng Chen,^1,2,3,†^

^1^National Laboratory of Solid State Microstructures & Department of Materials Science and Engineering, Nanjing University, Nanjing 210093, China

^2^Collaborative Innovation Center of Advanced Microstructures, Nanjing University, Nanjing 210093, China

^3^Jiangsu Key Laboratory of Artificial Functional Materials, Nanjing University, Nanjing 210093, China

**CONTENTS**

**I. Other topological phases** 2

**II. Tight-binding model** 3

**III. 1^st^-order surface states** 5

**III-I. Wilson loops** 5

**III-II. Fragile topology** 6

**IV. Tunable surface states** 8

**IV-I. Top and bottom facets** 8

**IV-II. Lateral facet** 9

**V. Lowering *C_4_* down to *C_2_*** 10

**V-I. Top facet** 10

**V-II. Lateral domain wall** 11

**VI. 2^nd^-order [001] hinge state** 12

**VI-I**. **Nested Wilson loop** 12

**VI-II. Hinge state and filling anomaly** 12

**VII. Other topological hinge states** 14

**VII-I. Hierarchical hinge arcs** 14

**VII-II. Surface valley-induced hinge state** 14

**VIII. 3^nd^-order corner state** 15

**IX. Extremal transmission of quadratic surface states** 16

**X. Robustness of multiple overlapping topological states again disorders** 17

**XI. Note on the losses** 20

**XI-I. Influence on the transport of sound energy** 20

**XI-II. Influence on the response spectra for corner state** 21

**XII. The localization lengths of various boundary modes** 23

**Experimental setup** 24

**I. Other topological phases**

| 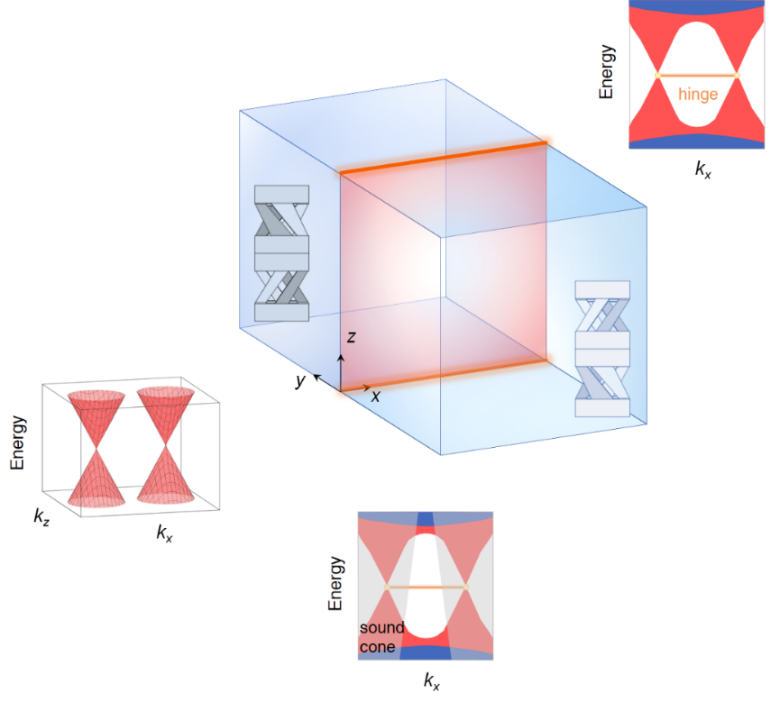 |
| --- |
| **FIG. S1. Schematics of Jackiw-Rebbi states on lateral domain wall.** Two surface Dirac cones exist on (010) domain wall [or equivalent (100) domain wall] between two oppositely placed phononic crystals. Further projection to the upper (reflection) or lower (radiation) hinge, the hierarchical 1D hinge arcs related to 2D surface Dirac nodes appear. See details in Fig. S9. |

| 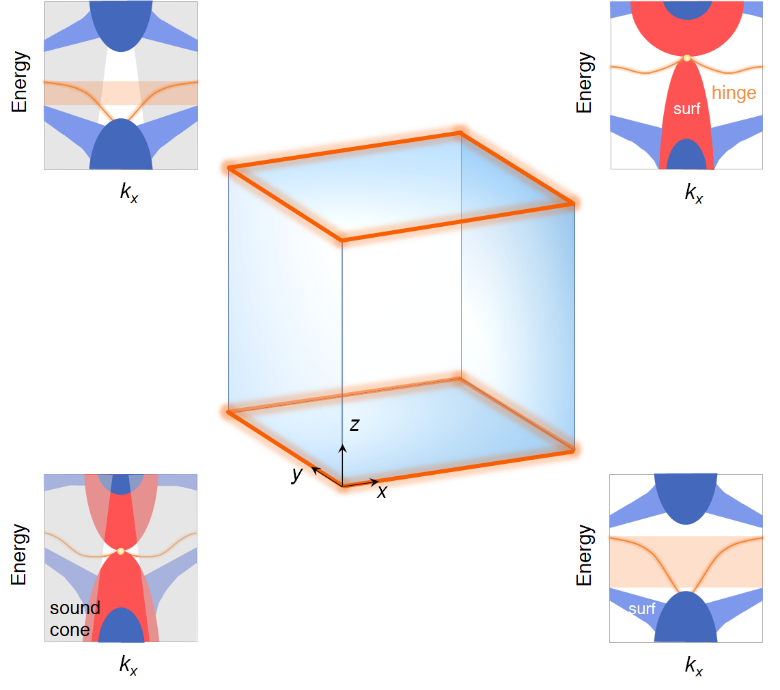 |
| --- |
| **FIG. S2. Schematics of the [100] or equivalent [010] hinge cases.** The 2D surface quadratic node on (001) or $\left( 00\bar{1} \right)$ facet can hierarchically support 1D hinge arc as well (upper-right and lower-left panels). After gapping the surface state, the valley-induced 2^nd^-order hinge arcs appear (upper-left and lower-right panels). See details in Fig. S11 and Fig. S12. |

**II. Tight-binding model**

| 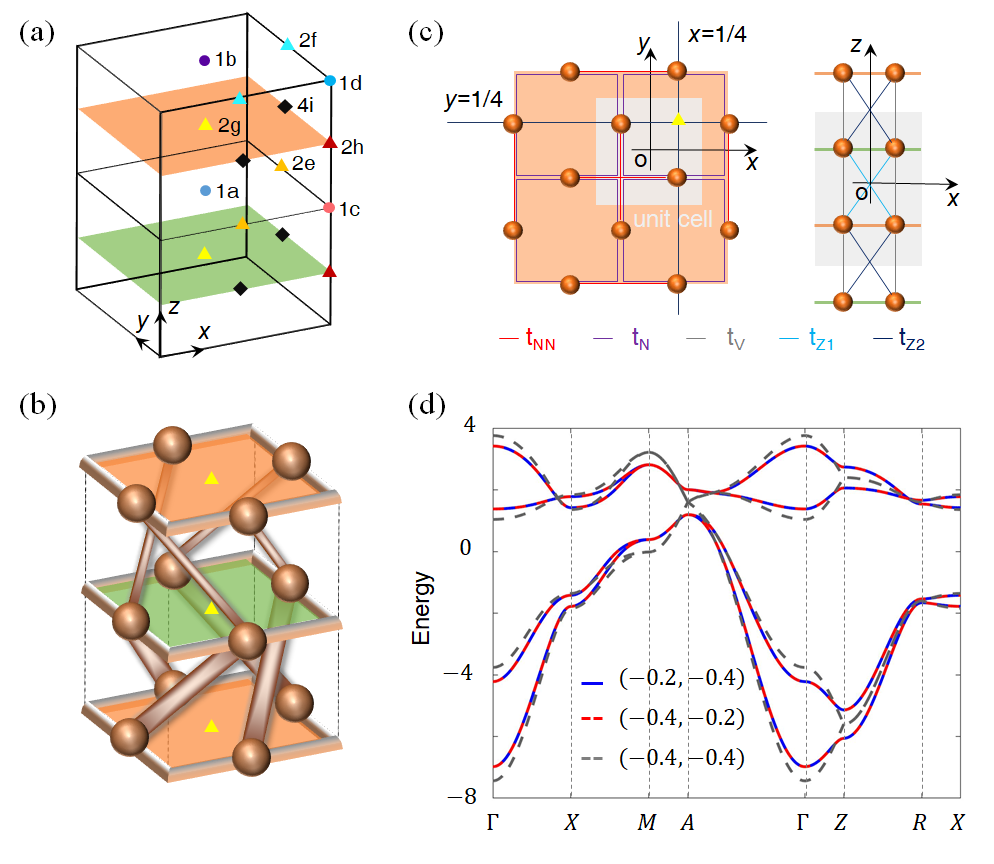 |
| --- |
| **FIG. S3. Band structures based on tight-binding model.** (a) Wyckoff positions of space group *P*422. (b) Tight-binding lattice model. (c) Schematics of both intra-layer couplings ($t_{N}$,$t_{NN}$) and inter-layer couplings ($t_{Z1}$, $t_{Z2}$,$t_{v}$). (d) Calculated band structure. The numbers in parentheses denote the inter-layer coupling strength (*t*_z1_, *t*_z2_). The other parameters: $t_{N}=-1$, $t_{NN}=-0.8$, and $t_{v}=0.3$ are the same. |

The basic lattice of space group *P*422 (No.89) belongs to a tetragonal system with three primitive lattice vectors: $\boldsymbol{a}_{1}=(a,0,0)$, $\boldsymbol{a}_{2}=(0,a,0)$, and $\boldsymbol{a}_{3}=(0,0,h)$. Here, we set $a$=1 for simplicity. There are four elementary symmetry generators, *i.e.*, *E*, *C_2z_*, *C_4z_*, and *C_2x/y_*. The Wyckoff positions of *P*422 are presented In Fig. S3(a), where the origin (1a) is set at the intersection of *C_4z_*, *C_2x_*, and *C_2y_* rotation axes. Here, 1a, 1b, 1c, 1d, 2e, and 2f are maximal Wyckoff positions, whose site-symmetry group is a maximal subgroup of the space group. In addition, we also plot parts of the non-maximal Wyckoff positions: 2g, 2h, and 4i.

To calculate the bulk band structures, we construct a tight-binding lattice model, as shown in Fig. S3(b)-(c). In one unit cell, there are four sites (two orange balls in two layers) located at 4i position:$\left( 0,1/2,1/2 \right)$, $\left( 1/2,0,1/2 \right)$,$\left( 0,1/2,-1/2 \right)$, and $\left( 1/2,0,-1/2 \right)$. In each layer (green and orange planes), the vertical tubes connecting neighboring sites act as either nearest-neighbor hopping ($t_{N}$) or next-nearest-neighbor hopping ${(t}_{NN})$. Between two layers, the opposite chirality of spiral tubes connecting two sites act as different inter-layer coupling ($t_{Z1}$ and $t_{Z2})$. Consequently, the Hamiltonian can be described as

$H\left( k \right)=\left[ \begin{matrix} \begin{matrix} h_{11} & h_{12} \\ h_{12}^{*} & h_{22} \end{matrix} & \begin{matrix} h_{13} & h_{14} \\ h_{23} & h_{13} \end{matrix} \\ \begin{matrix} h_{13}^{*} & h_{23}^{*} \\ h_{14}^{*} & h_{24}^{*} \end{matrix} & \begin{matrix} h_{11} & h_{12} \\ h_{12}^{*} & h_{22} \end{matrix} \end{matrix} \right]$, (S1)

where

$h_{11}=2t_{NN}\cos(k_{y})$,

$h_{22}=2t_{NN}\cos(k_{x})$,

$h_{12}=t_{N}(1+$ $e^{-ik_{x}}+e^{ik_{y}}+e^{-ik_{x}+ik_{y}})$,

$h_{13}={t_{v}(t}_{Z1}+t_{Z2}e^{ik_{z}h})$,

$h_{14}={(t}_{Z1}+t_{Z2}e^{ik_{z}h})*(e^{ik_{y}}+e^{-ik_{x}})$,

$h_{23}={(t}_{Z1}+t_{Z2}e^{ik_{z}h})*(1+e^{ikx-ik_{y}})$.

It should be noticed that two vertical inter-layer couplings with hopping strength (${t_{v}t}_{Z1}$ and ${t_{v}t}_{Z2}$) are considered to break the accidental degeneracies in BZ boundary [33], *e.g.*, the high-symmetry line *RX*. As shown in Fig. S3(d), a four-fold degeneracy protected by *z*-axis mirror symmetry emerge in *A* point. Such degeneracy can be gapped to construct a complete bulk bandgap by breaking the mirror symmetry such as $t_{Z1}\neq t_{Z2}$ (blue and red dashed lines). The case of |*t*_z1_|<|*t*_z2_| with gapped band structures match well with Fig. 2(d) in the main text.

**III. 1^st^-order surface states**

**III-I. Wilson loops**

| 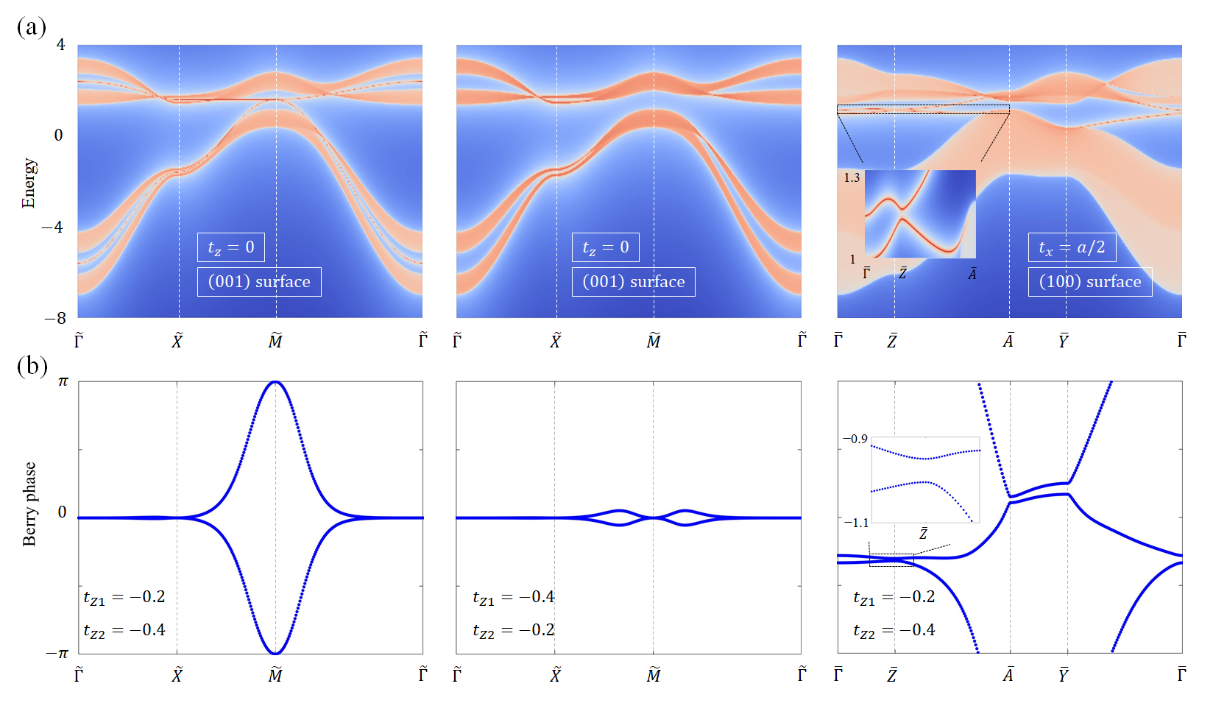 |
| --- |
| **FIG. S4. Surface spectra and Berry phases.** (a) Calculated projected band structures under different boundary conditions. Color scale represents the surface spectra density. (b) The corresponding Berry phases (Wilson loops). |

We use the tight-binding Hamiltonian (S1) to calculate the surface states under different boundary condition: (001), (100) facets for the case of |*t*_z1_|<|*t*_z2_| (corresponding to *w*_1_>*w*_2_ in the main text), and (001) facet for the case of |*t*_z1_|>|*t*_z2_| (corresponding to *w*_1_<*w*_2_). In Fig. S4(a), we show the surface spectral density $A\left( k,\omega\right)=-1/\pi\mathrm{Im}1/{(\omega-H\left( k \right)+i0)}$ along high-symmetry lines, which can be numerically obtained by iterative Green function. On the (001) facet, there exist quadratic surface states with topologically nontrivial nature for |*t*_z1_|<|*t*_z2_| (left panel). In contrast, for the case of |*t*_z1_|>|*t*_z2_| (middle panel), no in-gap surface states are observed.

To analyze the difference, we resort to the hybrid Wannier centers $\bar{z}_{n}\left( k_{\perp} \right)$, or saying non-Abelian Berry phase $\varphi_{n}\left( k_{\perp} \right)$ [39]. Here, *n* labels the index of bands, and $k_{\perp}$ is wave vector in the surface BZ. The multi-band non-Abelian Berry phase can be calculated through the eigenvalue problem of Wilson loop:

$W\left( k_{\perp} \right)\left| \left. v_{\perp} \right\rangle\right.=e^{i\varphi_{n}\left( k_{\perp} \right)}\left| \left. v_{\perp} \right\rangle\right.,$ (S2)

$W\left( k_{\perp} \right)=\prod M_{mn}^{k_{//},k_{//}+\triangle k},$ (S3)

where $M_{mn}^{k_{//},k_{//}+\triangle k}=\left\langle u_{mk_{//}} | u_{n(k_{//}+\triangle k)} \right\rangle$ is the linking matrices of two Bloch wavefunctions at two neighboring *k* points, and the product is carried out along the $k_{//}$ direction.

As shown in Fig. S4(b), in the case of |*t*_z1_|<|*t*_z2_| (left panel), the Berry phase winds the whole [-π π] range, indicating its nontrivial manner with gapless surface states. But, the Berry phase spectrum for the case of |*t*_z1_|>|*t*_z2_| facet shows trivial (middle panel). The phase transition occurs at |*t*_z2_|=|*t*_z1_| point, where the bulk band gap close [grey dashed lines in Fig. S3(d)]. Moreover, the lateral (100) facet is trivial as well, where the existence of gapped surface states without symmetry-enforced degeneracy is attributed to nonzero surface dipole moment (right panel).

**III-II. Fragile topology**

| 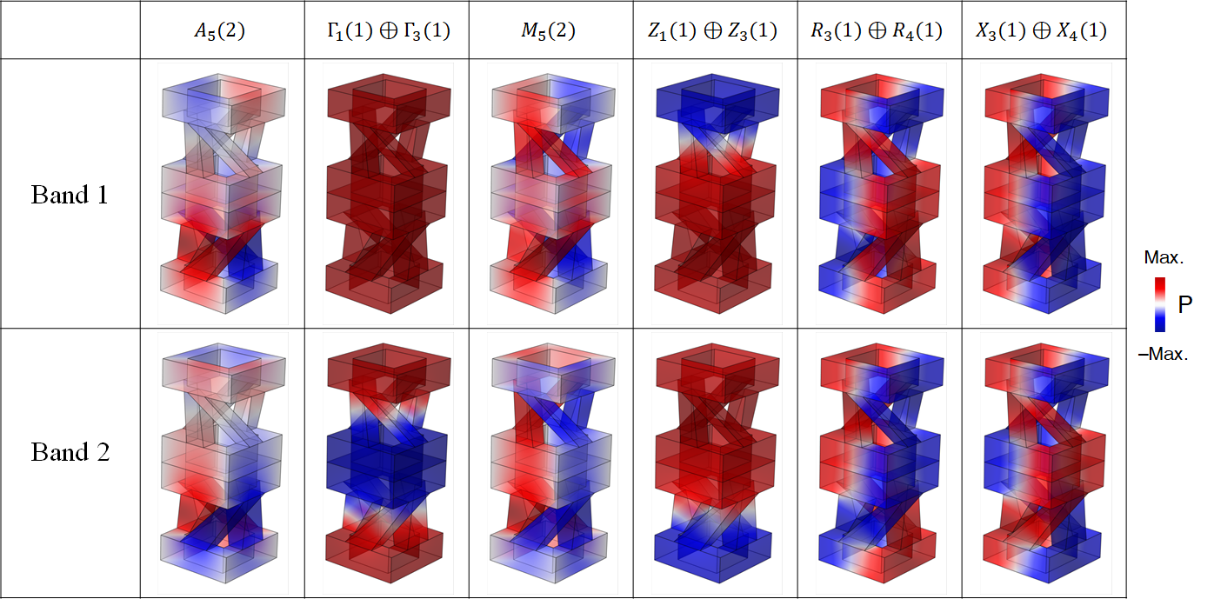 |
| --- |
| **FIG. S5. Acoustic field distributions at the maximal momenta of two lower bands**. The irreducible representations are denoted in the first row. |

In addition, we characterize the bulk topology via the method of elementary band representations (EBRs) analysis. EBRs are special sets of indecomposable band representations induced from the orbitals of the site-symmetry group at Wyckoff positions. The EBRs offer a simple yet strong tool to assess the topology of bands. For any band structure with symmetry data vector B, *i.e.*, collected multiplicities of irreducible representations at all maximal momenta, we can always find a linear combination of EBRs satisfying:

$B_{i}=\sum_{j} {[EBR]}_{ij}p_{j}$, (S4)

where the *p*-vectors directly gives the topological classification.

In our system, the acoustic field distributions at all maximal momenta of the lower two bands are given in Fig. S5, yielding the symmetry data vector as:

$B=\left( 0,0,0,0,1,1,0,1,0,0,0,0,0,0,1,1,0,1,0,0,0,0,1,1,0,0,1,1 \right)^{T}.$ (S5)

The coefficient is given in the order of:

$B=\left( m\left( A_{1-5} \right),m\left( \Gamma_{1-5} \right),m\left( M_{1-5} \right),m\left( Z_{1-5} \right),m\left( R_{1-4} \right),m\left( X_{1-4} \right) \right)^{T},$ (S6)

where $m\left( K_{i} \right)$ denotes the multiplicities of the *i^th^* irreducible representation of the little group at the maximal momenta *K*. In space group *P*422, there are 28 EBRs available on the Bilbao Crystallographic Server (BCS), and each EBR has 28 components. Therefore, the all symmetry information of space group *P*422 can be manifested in a 28×28 integer matrix *EBR*, which has a unique Smith normal form:

$EBR=L_{EBR}\Lambda_{EBR}R_{EBR}$. (S7)

Herein,$L_{EBR}$ is 28×28 unimodular integer matrix,$R_{EBR}$ is unimodular matrix and$L_{EBR}$ is the Smith normal form of *EBR* with its rank being 12. By solving the equations S4-S7, we can obtain the *p*-vectors in the form:

$p_{i}=\sum_{j=1}^{12} {[R_{EBR}^{-1}]}_{ij}\frac{1}{{[\Lambda_{EBR}]}_{ij}}{(L_{EBR}^{-1}B)}_{j}+\sum_{j=1}^{16} {[R_{EBR}^{-1}]}_{i,j+12}k_{j}$, (S8)

where $k_{1-16}$ are free integer parameters. By setting $k_{1-16}=0$, one *p*-vector is:

$p=\left( 0,0,-1,0,0,0,0,0,0,0,0,1,0,0,0,0,0,0,0,0,1,0,0,0,0,0,0,0 \right)^{T}$. (S9)

It implies that the lower two bands in our system can be decomposed as ${(A_{1})}_{2e}\bigoplus{(A_{2})}_{1c}⊖{(B_{1})}_{1a}$, where $⊖$ denote the missing EBR induced from Wyckoff position 1a and characterize the fragile topology of our system.

**IV. Tunable surface states**

**IV-I. Top and bottom facets**

| 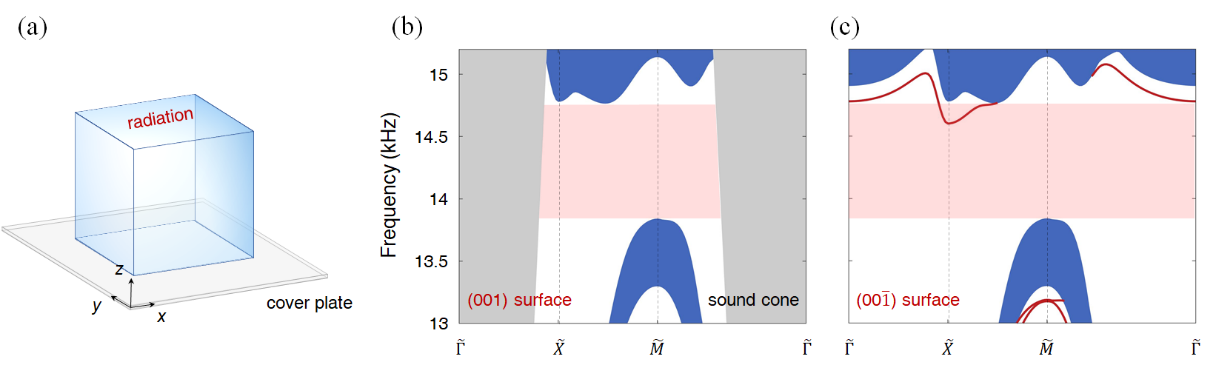 |
| --- |
| **FIG. S6. Removal of in-gap surface states**. (a) Schematic of sample with top radiation and bottom reflection (cover plate) facets. (b)-(c) Projected band structures of (001) and $(00\bar{1})$ surface states. In both cases, the surface terminations are *t_z_*=*s*. |

Due to the fragile behavior, the in-gap (001) surface states on the reflection top facet can be removed by adopting a radiation boundary, and vice versa for the $(00\bar{1})$ facet, as shown in Fig. S6. Besides the bulk topology, the existence of gapless surface states [Fig. 2(e) and Fig. 4(a) in the main text] also depends on the surface conditions as well, not stable as the electronic TI with unremovable surface states. However, this unstable character provides a convenient way to manipulate the acoustic waves by tuning the surface condition. For example, we can selectively turn on/off the acoustic signals on $(00\bar{1})$ facet by simply removing/covering a hard plate.

**IV-II. Lateral facet**

| 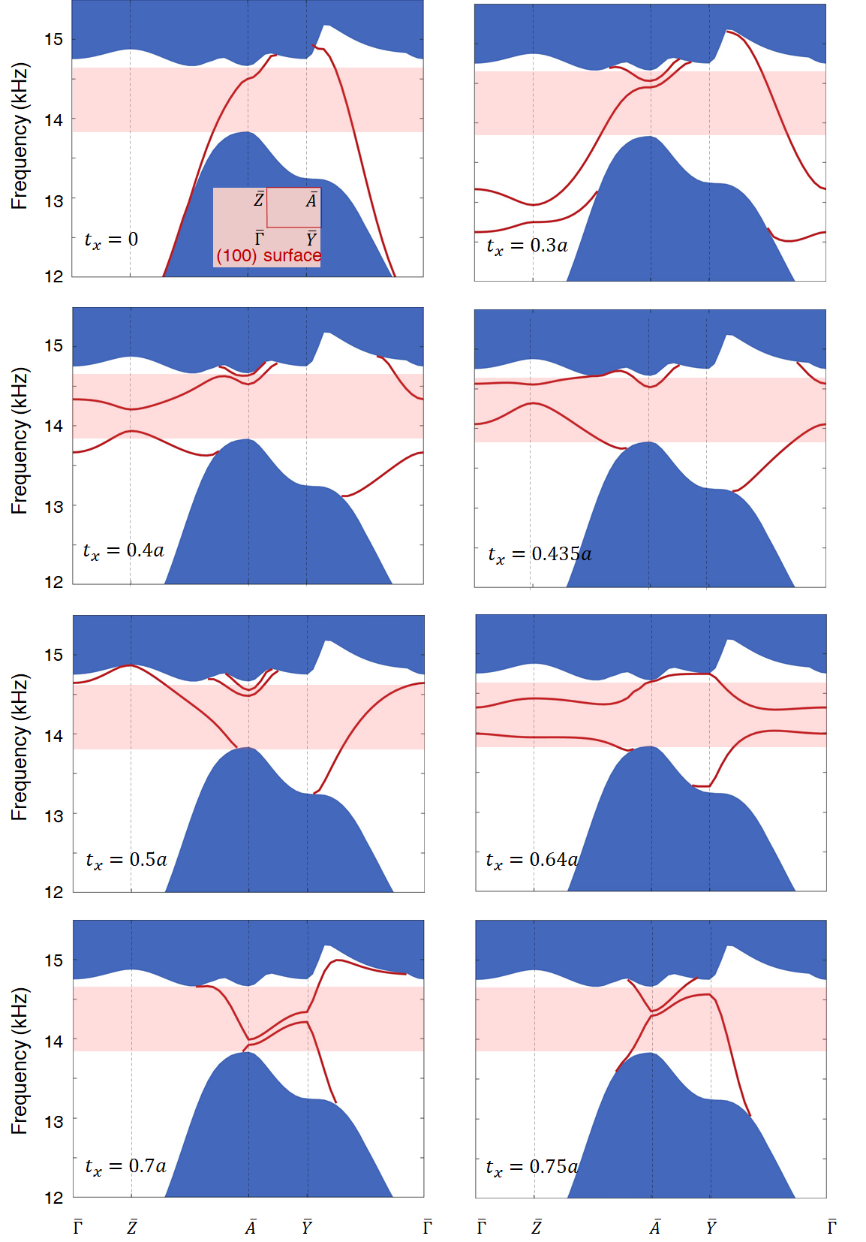 |
| --- |
| **FIG. S7. Tunable** $\mathbf{(100)}$ **surface states with various truncated parameter *t_x_*.** The red lines represent the gapped surface states. The red shadow regions denote the complete bulk bandgap. |

In contrast to the top or bottom facet with degenerated surface node protected by *C_4z_*, the lateral facets with *C_2x/y_* fail to keep degeneracy. However, the gap of surface states holds possibilities to support higher-order phases, *e.g.*, [001] hinge. Therefore, we can truncate the lateral facets to obtain a complete surface bandgap for the hinge state, as shown in Fig. S7. In experiments, we choose *t_x_*=0.435*a* on both lateral facets, measuring the hinge spectrum within the surface bandgap from 14.3 to 14.5 kHz [Fig. 5(c) in the main text]. Due to *C_4z_* rotation, the result is the same on the (010) facet case.

**V. Lowering *C_4_* down to *C_2_***

**V-I. Top facet**

| 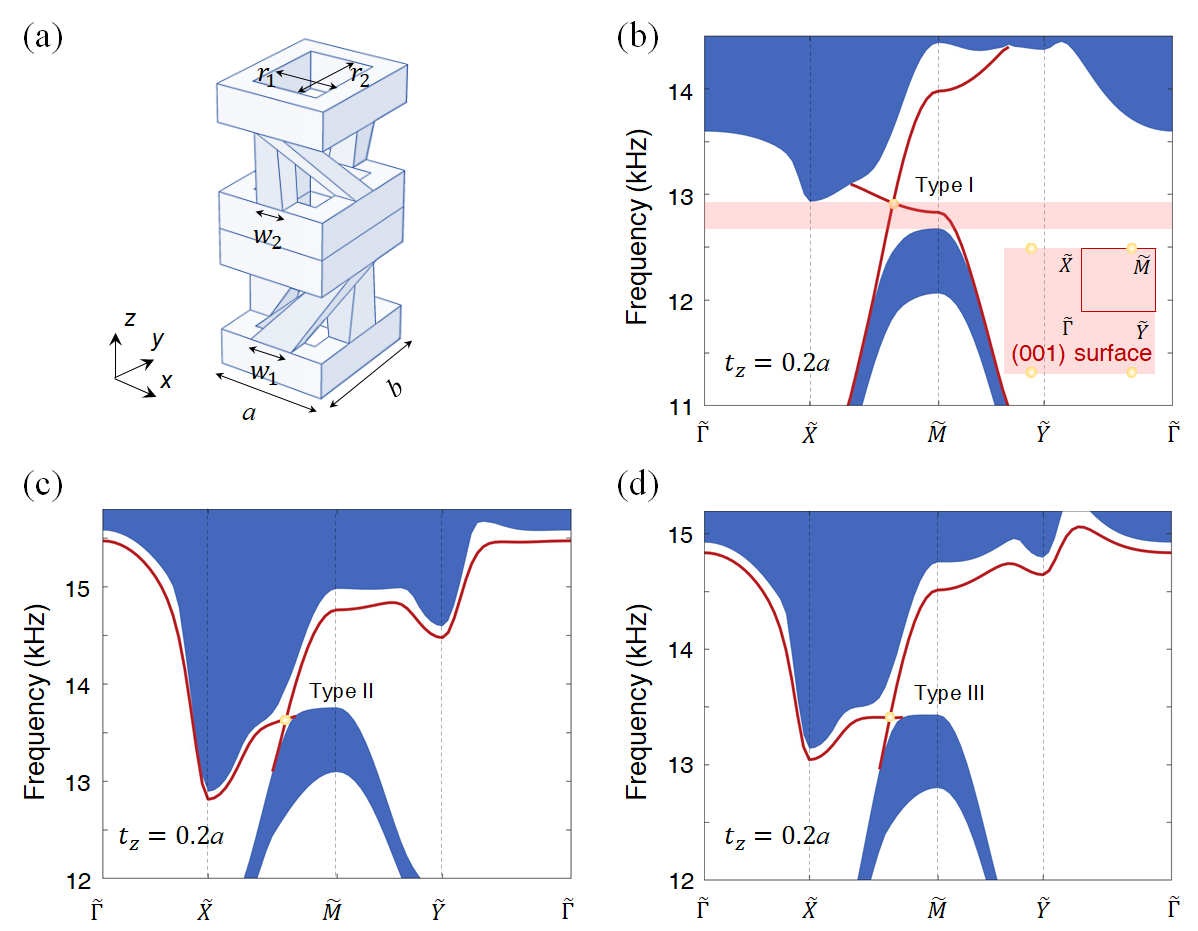 |
| --- |
| **FIG. S8. C_2_-invariant topological crystalline crystal with two surface Dirac cones.** (a) A unit cell of orthorhombic lattice characterized by *b*=1.2*a*. (b) Projected band structures with two type I surface Dirac cones. The parameters are *w*_1_=4 mm, *w*_2_=2.5 mm, *r*_1_=6 mm, and *r*_2_=8 mm. (c) Type II case with structure parameters: *w*_1_=3.5 mm, *w*_2_=2.5 mm, *r*_1_=4 mm, and *r*_2_=6 mm. (d) Type III case with structure parameters: *w*_1_=3.5 mm, *w*_2_=2.5 mm, *r*_1_=4.8 mm, and *r*_2_=6.8 mm. The other parameters are the same as those in the main text. |

The single quadratic degeneracy pinned to surface momenta $\tilde{M}$ is stabilized by both time-reversal and *C_4z_* rotation symmetry. Lowing *C_4z_* down to *C_2z_* then split the quadratic degeneracy into two surface Dirac nodes. To achieve this, we stretch the tetragonal lattice to an orthorhombic one with its lattice constant satisfying *b*=1.2*a* [Fig. S8(a)]. The projected band structures are plotted in Fig. S8B-D, where two surface Dirac cones appear along the high-symmetry line $\tilde{M}\tilde{X}$. The Dirac cones with a point-like Fermi surface resemble type I case [Fig. S8(b)]. Moreover, through tuning the structure parameters, the surface Dirac cones can be tilted into either type II case [Fig. S8(c)] with an arc-like Fermi surface or type III [Fig. S8(d)] with a line-like one, further broadening the phase category of our model.

**V-II. Lateral domain wall**

| 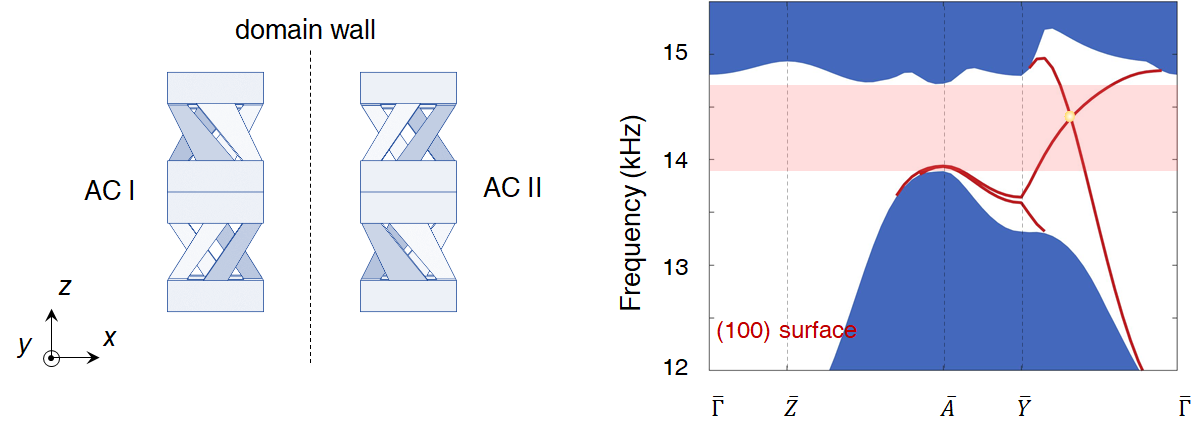 |
| --- |
| **FIG. S9. The Jackiw-Rebbi states on (100) domain wall.** Two surface Dirac cones appear at the lateral domain wall between two oppositely placed the exact structure of opposite effective mass (AC I and AC II). |

For lateral facets with *C_2x/y_* rotation, the surface Dirac cones can also be realized on a domain wall configuration. As shown in Fig. S9, the domain wall configuration is constructed via two *z*-axis oppositely placed phononic crystals (or relatively half-a-lattice shift along the *z* direction). These two phononic crystals possess opposite effective mass, whose interface supports Jackiw-Rebbi states. In this sense, our structure resembles a classical-wave analogue of dual TI, with both a weak TI phase (in terms of even surface Dirac cones) and a TCI phase (in terms of single surface quadratic degeneracy protected by crystalline symmetry).

**VI. 2^nd^-order [001] hinge state**

**VI-I**. **Nested Wilson loop**

| 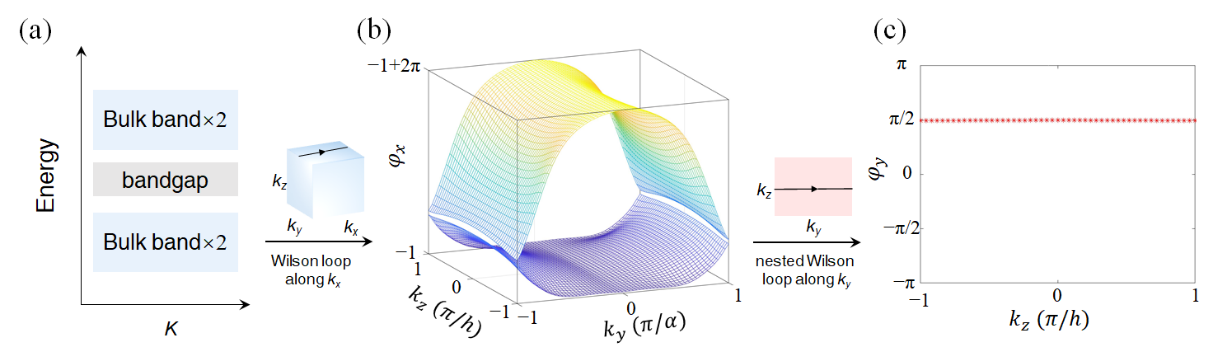 |
| --- |
| **FIG. S10. Nested Wilson loop.** (a) Illustration of a Wilson loop along the *k_x_* direction for bulk bands. (b) Nested Wilson loop along the *k_y_* direction for 2D Wannier bands. (c) Calculated 1D Wannier bands along the *k_z_* direction. |

In this section, we use the nested Wilson loop method to characterize the 2^nd^-order [001] hinge state, which is wildly used to explain higher-order topological phases. First, we calculate a Wilson loop along the *x* direction for the two bulk bands below the bandgap, yielding the distributions of hybrid Wannier centers on 2D *k_y_k_z_*-surface BZ (Wannier bands). As shown in Fig. S10(b), this procedure splits the two degenerated bands into two gapped Wannier bands. Being gapped, we can define the Wannier band bias in each sector (denote as + or $-$) as: $\left| \left. w_{\pm} \right\rangle\right.=\sum_{n} \left| \left. u_{nk_{y}} \right\rangle\right.{[v_{k_{y},x}^{\pm}]}^{n}$, where ${[v_{k_{y},x}^{\pm}]}^{n}$is the *n*^th^ component of the eigenfunction of the *x*-direction Wilson loop. Then, we calculate a second *y*-direction Wilson loop. Herein, the lower Wannier band is chosen, yielding the nested Berry phase $\pi/2$ for all *k_z_*-slices. It shows the charge center located at *y*=1/4. Thus, when the sample termination is identical to the charge center [yellow triangles in Fig. S3(c)], there will find hinge states.

**VI-II. Hinge state and filling anomaly**

In a dimension reduce procedure, our structure can be viewed as a collection of 2D gapped systems parametrized by *k_z_*. In each *k_z_*-slice, the *C*_4_*_z_* rotation keeps intact. The presence of *C*_4_*_z_* symmetry, therefore, allows for a classification of 2D gapped systems in terms of rotation topological invariants, which are related to the symmetry representation at high-symmetry points of bulk BZ [34]. These topological invariants are given as:

$\chi^{4}=(\left[ X_{1}^{\left( 2 \right)} \right],\left[ M_{1}^{\left( 4 \right)} \right],\left[ M_{2}^{\left( 4 \right)} \right])$. (S10)

$\left[ \Pi_{p}^{\left( n \right)} \right]\equiv⋕\Pi_{p}^{\left( n \right)}-⋕\Gamma_{p}^{\left( n \right)}, \Pi=X,M$, count the difference between the number of eigenstates with rotation eigenvalue $e^{2\pi i(p-1)/n}$ at high-symmetry point $\Pi$ and those at $\Gamma$. Through examining the symmetry properties at $\Gamma,X,M$ points in $k_{z}=0$ slice and $Z,R,A$ points in $k_{z}=\pi/h$ slice (Fig. S5), we can obtain $\chi^{4}=(-2,-2,1)$. The results are also applied to other *k_z_*-slices. Therefore, the nontrivial topological index leads to quantized corner charge:

$Q=\frac{1}{4}\left( [X_{1}^{\left( 2 \right)}]+{2[M}_{1}^{\left( 4 \right)}]+3{[M}_{2}^{\left( 4 \right)}] \right) \mathrm{mod} 1=\frac{1}{4}$. (S11)

The nonzero fractional quantization of corner charge arises from a filling anomaly between *C*_4z_ symmetry and the filling number of particles in the lattice, leading to strongly localized corner modes. As a consequence, our 3D structure stacked by 2D *k_z_*-dependent slices can support localized hinge states on a finite sample originating from fractional quantized hinge charge.

**VII. Other topological hinge states**

**VII-I. Hierarchical hinge arcs**

| 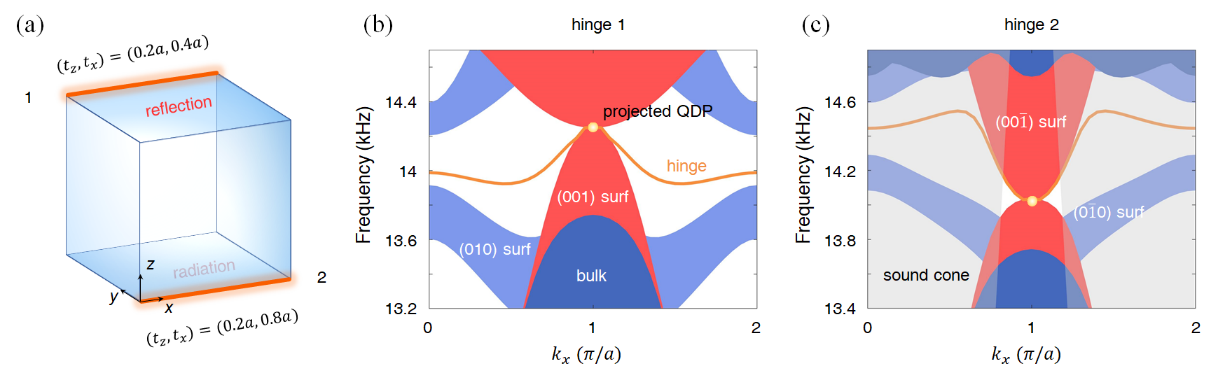 |
| --- |
| **FIG. S11. [100] hinge arcs attached to the surface quadratic surface node.** (a) Schematic of our cuboid with reflection hinge 1 and radiation hinge 2. Parameters *t_z_* and *t_x_* denote the truncated top (or bottom) and lateral facets. (b)-(c) Hinge spectra corresponding to hinge 1 and hinge 2. In both cases, the hinge arcs connect the projection of the surface quadratic degenerated point (QDP). These hierarchical cases are similar to 2D Dirac case with 1D edge arcs connecting to bulk Dirac nodes. |

**VII-II. Surface valley-induced hinge state**

| 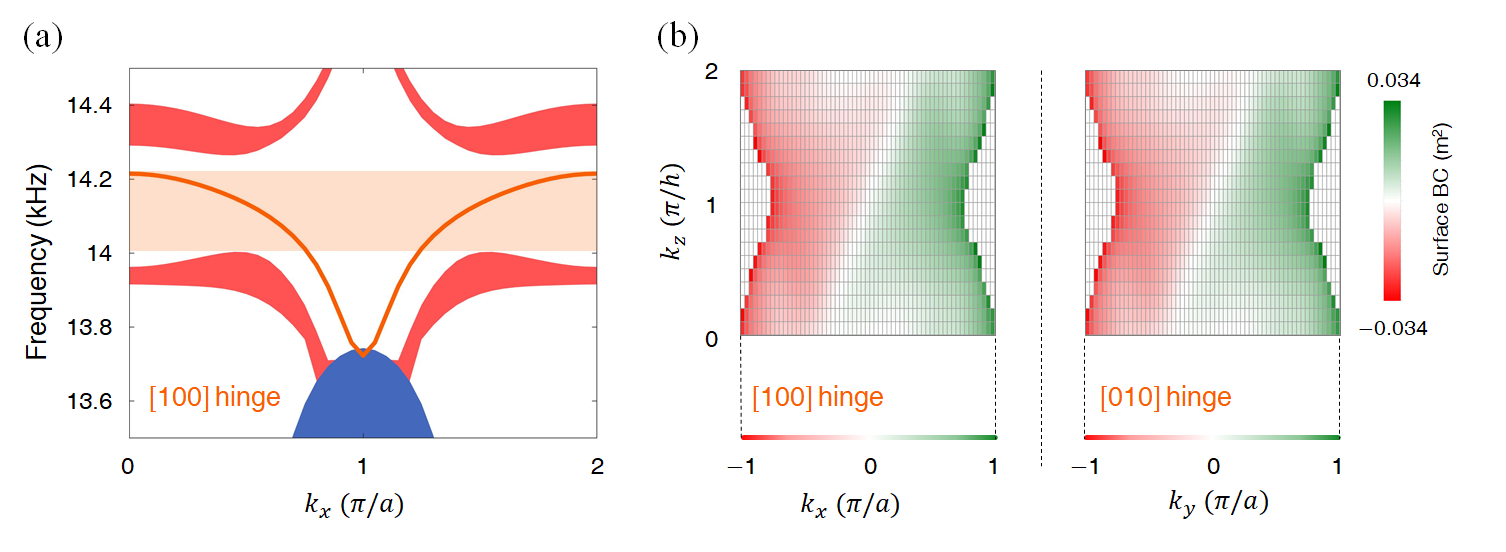 |
| --- |
| **FIG. S12. Hinge spectrum and surface Berry curvature.** (a) [100] hinge spectrum with reflection hinge (*t_z_*=*s*, *t_x_*=0.64*a*). (b) Surface Berry curvatures of the lower surface state on (100) facet (left panel) and (010) facet (right panel). Near the surface BZ boundary, the surface Berry curvatures are not well-defined due to the hybrid between surface states and bulk states. These gapped lateral facets manifest opposite surface Berry curvatures in two halves of BZ that can induce valley-like hinge states. |

**VIII. 3^nd^-order corner state**

| 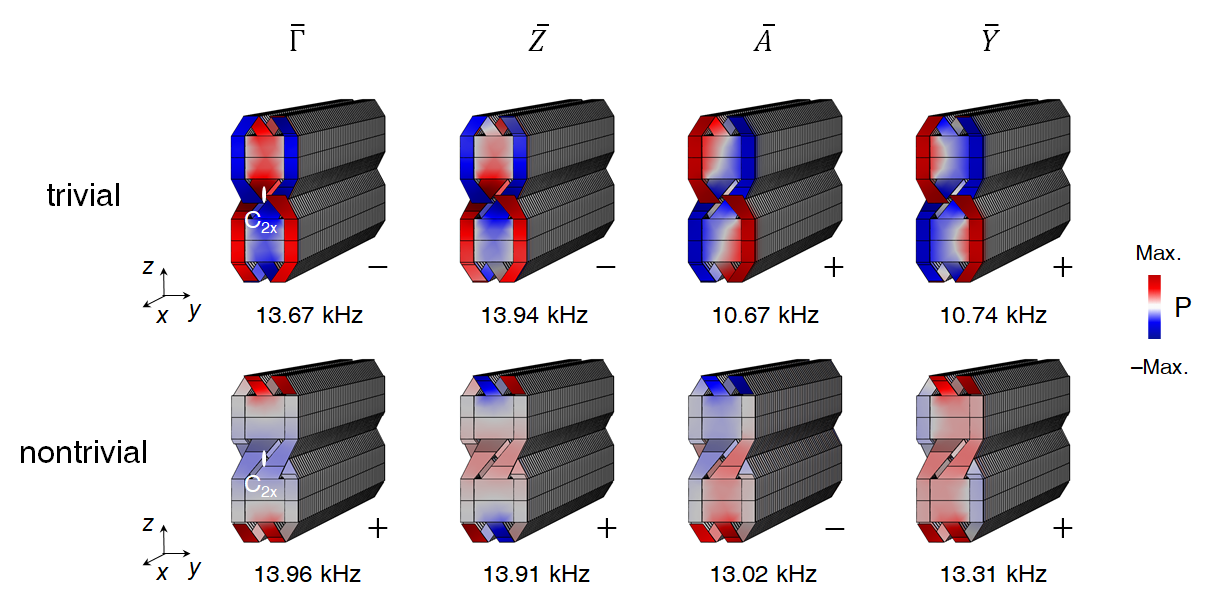 |
| --- |
| **FIG. S13. Acoustic field distributions of lateral facets** at the maximal surface momenta of the lower (100) surface band, where the + (−) denote the symmetric (anti-symmetric) field respective to the *C*_2_*_x_* rotation axis. The trivial and nontrivial cases correspond to truncated cases *t_x_*=0.4*a* and *t_x_*=0.64*a*, respectively. |

In experiments, the corner is fabricated as the intersection of three facets with hard wall boundaries. The truncated (100), (010), and ($00\bar{1}$) facets are *t_x_*=0.64*a*, *t_x_*=0.64*a*, and *t_z_*=*s*, respectively. The corner state is attributed to higher-order topology of the lateral (100) or (010) facet with *C*_2_ rotation. We focus on the (100) facet (*t_x_*=0.64*a*) and consider a supercell configuration. Thus, the (100) facet with localized decaying surface states can be viewed as a 2D model, and thus can be classified into distinct phases in terms of *C*_2_-rotation topological invariants [34],

$\chi^{2}=(\left[ \bar{Z}_{1}^{\left( 2 \right)} \right],\left[ \bar{Y}_{1}^{\left( 2 \right)} \right],\left[ \bar{A}_{1}^{\left( 2 \right)} \right])$. (S12)

$\left[ \bar{\Pi}_{1}^{\left( 2 \right)} \right]\equiv⋕\bar{\Pi}_{1}^{\left( 2 \right)}-⋕\bar{\Gamma}_{1}^{\left( 2 \right)}, \Pi=Z,Y,A$, counts the difference between the number of *C_2_*-symmetry eigenstates at high-symmetry surface momenta $\bar{\Pi}$ and those at $\bar{\Gamma}$. The distributions of surface states at high-symmetry point $\bar{\Gamma}$, $\bar{Z}$, $\bar{A}$ and $\bar{Y}$ (Fig. S13) give rise to a nontrivial index $\chi^{2}=(0,0,-1)$, leading to the fractional corner charge,

$Q=\frac{1}{4}\left( -[\bar{Z}_{1}^{\left( 2 \right)}]-[\bar{Y}_{1}^{\left( 2 \right)}]+[\bar{A}_{1}^{\left( 2 \right)}] \right) \mathrm{mod} 1=\frac{3}{4}$. (S13)

For the trivial case (*t_x_*=0.4*a*), the index $\chi^{2}=(0,1,1)$ gives Q=0, corresponding to the absence of corner states. The transition between trivial (*t_x_*=0.4*a*) and nontrivial case (*t_x_*=0.64*a*) is approximately located at *t_x_*=0.5*a*, where the surface bands get degenerated with bulk bands (Fig. S7).

**IX. Extremal transmission of quadratic surface states**

| 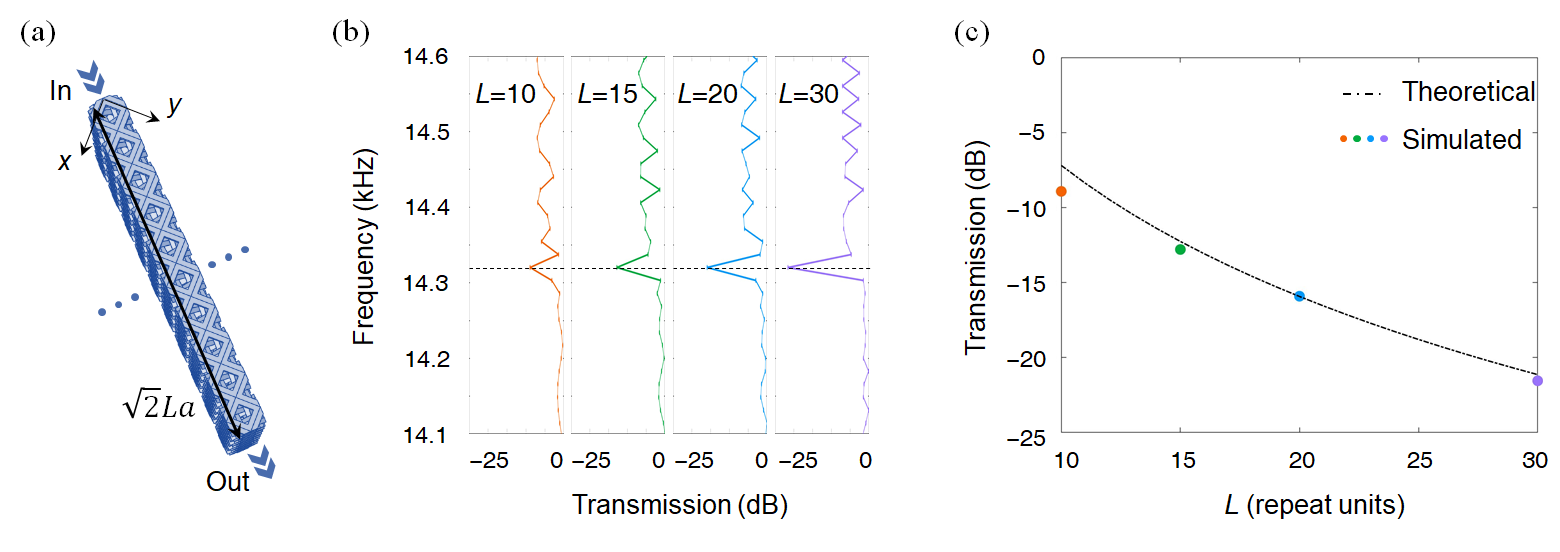 |
| --- |
| **FIG. S14. Extremal transmission of the quadratic surface states.** (a) Illustration of supercell configuration to calculate transmission on (001) facet. The wave is incident from [110] direction. (b) Simulated transmission spectra at various lengths of unit cells (*L*). (c) Relation between transmission and length near the frequency of quadratic surface node*.* The color dots are numerical results, and the black dotted line is the theoretical result. |

To theoretically investigate the relation between transmission and length, we use an effective 2D Hamiltonian to describe the quadratic degenerated surface bands:

$H=\left[ \begin{matrix} {t_{I}(k_{u}^{2}-k_{v}^{2})+t}_{z}(k_{u}^{2}-k_{v}^{2}) & 2t_{x}k_{u}k_{v} \\ 2t_{x}k_{u}k_{v} & {t_{I}(k_{u}^{2}-k_{v}^{2})-t}_{z}(k_{u}^{2}-k_{v}^{2}) \end{matrix} \right]$, (S14)

where $k_{u}$ and $k_{v}$ are displacement from the node. For simplicity, we set $t_{I}=0$ and $\left| t_{x} \right|=\left| t_{z} \right|$.

By applying continuous boundary conditions, for the *n*^th^ mode of wave function inside the sample, we can get the transmission coefficient:

$t_{n}=\frac{2i\tilde{k}k_{l}}{-\left( \tilde{k}^{2}+k_{l}^{2} \right)\sin\left( \tilde{k}L \right)+2i\tilde{k}k_{l}\cos\left( \tilde{k}L \right)}$, (S15)

where $\tilde{k}$ and $k_{l}$ are inside and outside wave vectors. Near the degeneracy, we estimate $k_{l}\sim\sqrt{U}\gg\tilde{k}$, *U* is a constant, and $\tilde{k}=\pm iq_{n}$ to calculate the transmission probability:

$T(q_{n})={|t_{n}|}^{2}=\frac{4}{4\cosh^{2}\left( q_{n}L \right)+{U\sinh^{2}\left( q_{n}L \right)}/{q_{n}^{2}}}$, (S16)

where $q_{n}={2n\pi}/W, n\in Z$, and *W* denotes the wideness of sample ($W\gg L$). Particularly, we can get $\lim_{q_{n}\to0} T\left( q_{n} \right)=\frac{4}{(4+UL^{2})}$. Then, every individual mode contributes to the total transmission:

$T\propto\sum_{n=-\infty}^{n=+\infty} T\left( q_{n} \right).$ (S17)

In Fig. S14(c), we plot the numerical result (black dotted line) with parameters: *W*=100, *U*=1. Compared to *L*^-1^-related extremal transmission at linear Dirac case [40], the transmission near the quadratic degeneracy is approximately related to *L*^-2^.

**X. Robustness of multiple overlapping topological states again disorders**

| 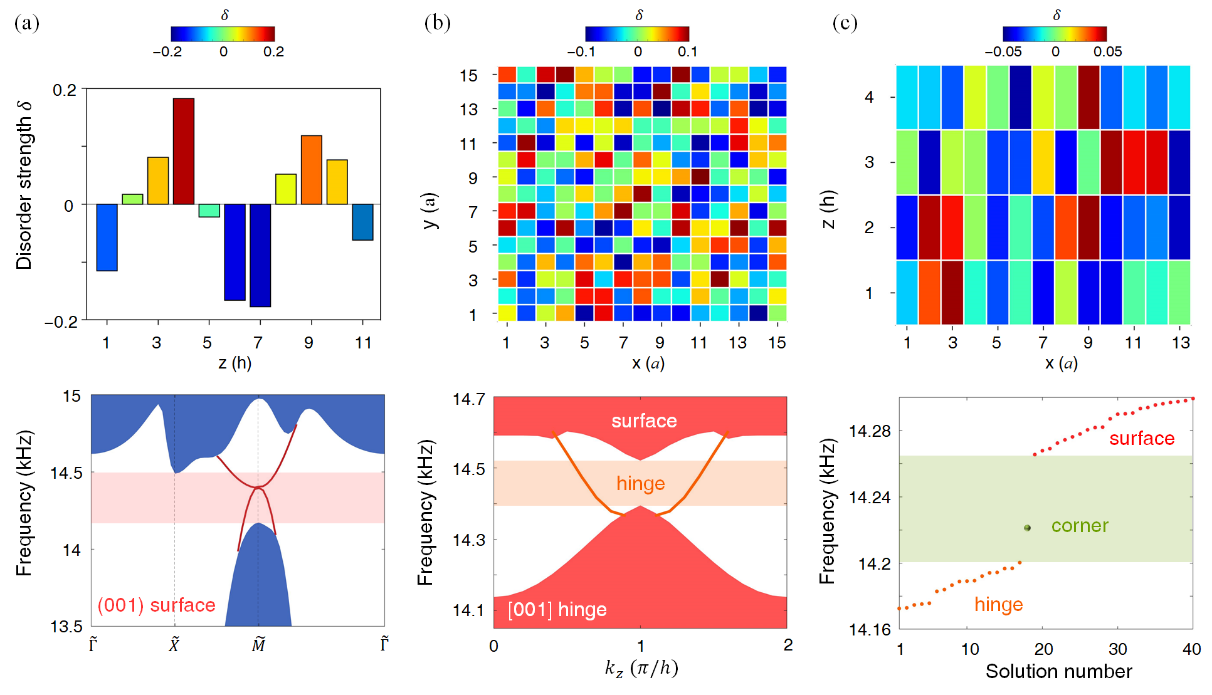 |
| --- |
| **FIG. S15. Robustness of topological boundary states against random perturbations.** (a) The (001) surface states (bottom panel) of a *z*-directed supercell with 11 randomly distributed coupling strengths (top panel). Such disorders keep C_4_*_z_* symmetry to guarantee quadratic degeneracy. (b) The [001] hinge state of a 15×15 supercell with random couplings. (c) The corner state with a 13×13×4 structure, where the disorders are introduced to the *xz* facet. Compared to intact lattice cases in the main text, the boundary states are still maintained. |
| 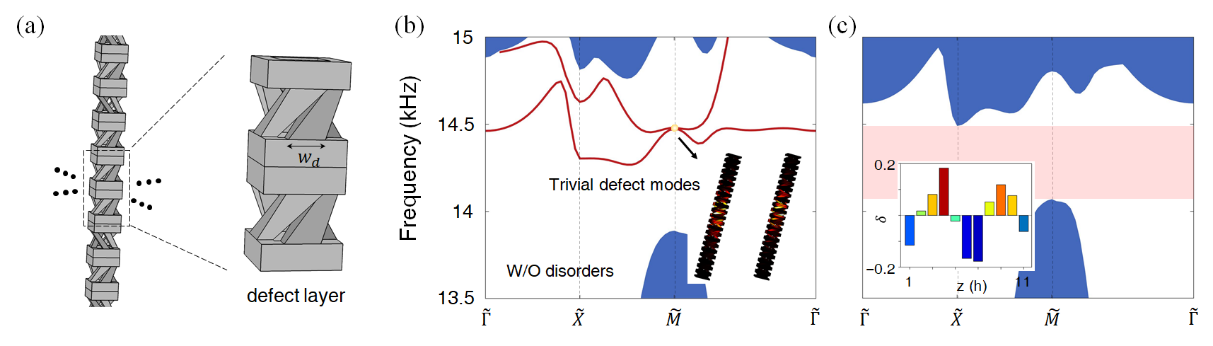 |
| **FIG. S16. Unstable trivial waveguide case.** (a) Waveguide via defect layer structure composed of two identical chiral tubes with *W_d_*=0.45*a*. (b) Trivial surface states with a pair of in-gap defect modes. (c) The surface states completely disappear under the *z*-directed disorder perturbation [inset, the same as those in Fig. S15(a)]. |

To study the robustness again disorders, we modulate the width of chiral tubes (corresponding to inter-layer coupling strength in the TBA model) as *w*_1,2_(1+δ), where δ represents the disorder strength. For 2D surface states, a *z*-directed supercell with 11 randomly distributed coupling strengths is constructed, where δ ranges from [−0.2 0.2]. Here, we assume these perturbations are uniform in each layer. Thus, the C_4_*_z_* rotation symmetry is unchanged, which can still keep the quadratic degeneracy of surface states. As shown in Fig. S15(a), the gapless quadratic surface states are robust against disorders ($\pm$20% tolerance) as long as the bulk bandgap survives. Similar to the z-hinge state using a 15×15 supercell structure (δ ranging from [−0.1 0.1], $\pm$10% tolerance) [Fig. S15(b)] and the corner state using a 13×13×4 structure (δ ranging from [−0.05 0.05], $\pm$5% tolerance) [Fig. S15(c)]. In the corner configuration, the disorders are introduced to the *xz* facet and chiral tubes *w*_2_. The tolerances for robust surface, hinge, and corner states against disorders decrease with the decreased bandgaps.

As a comparison, we design a trivial waveguide case by introducing a defect layer composed of two identical chiral tubes [Fig. S16(a)]. The parameter (*w_d_*=0.45*a*) is finely tuned to ensure the trivial defect modes are located within the bandgap [Fig. S16(b)]. After introducing the same *z*-directed disorder strength in Fig. S15(a), the trivial defect modes no longer survive [Fig. S16(c)]. In contrast, the boundary modes induced from nontrivial topology are less sensitive to local disorders than trivial ones. Similar protections also work for the cases of the hinge and corner states.

| 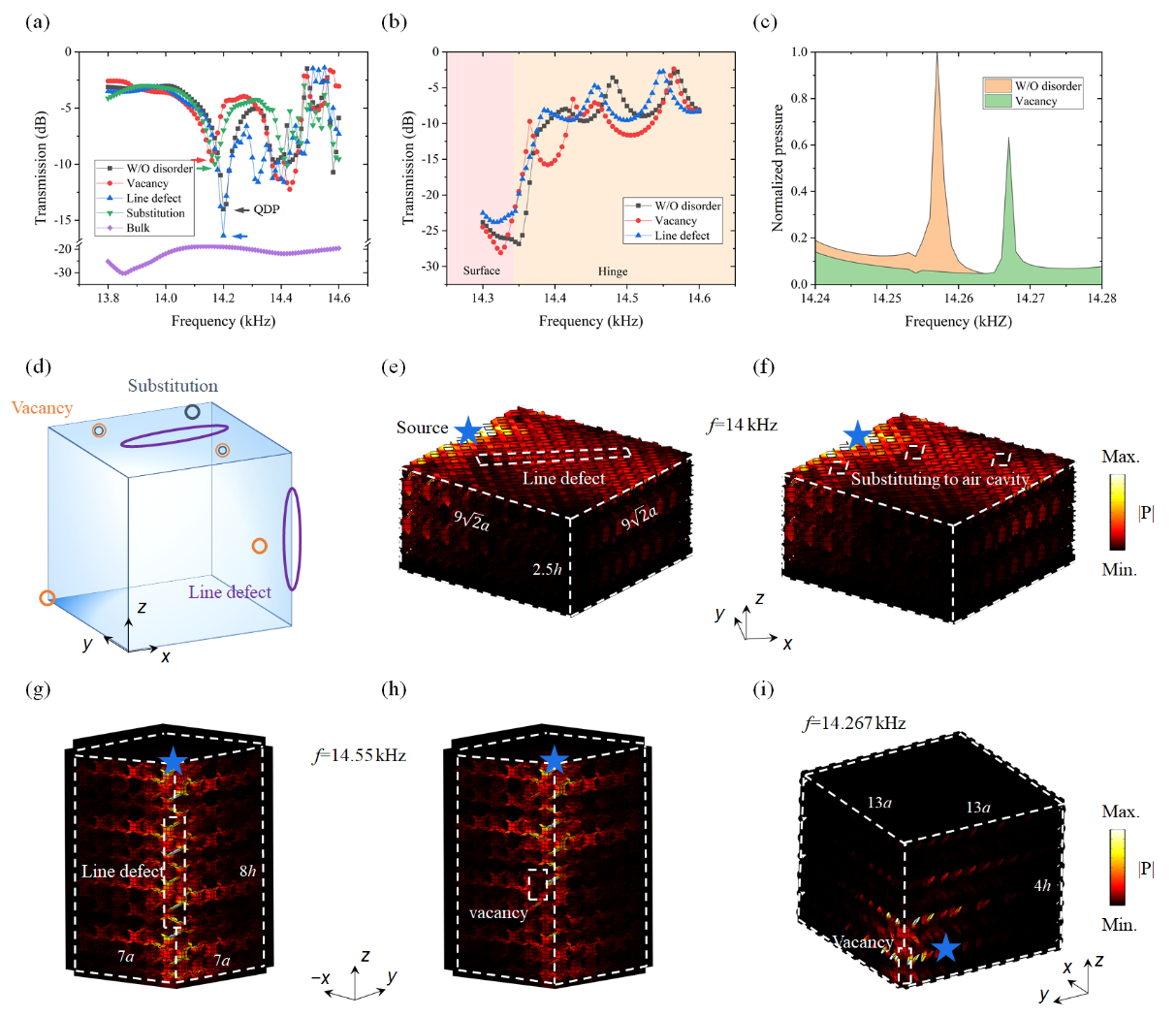 |
| --- |
| **FIG. S17. Robustness of multiple boundary states under various defects.** (a) Simulated bulk (purple line) and $\tilde{\Gamma}\tilde{M}$ surface (other color lines) transmission spectra. The quadratic degenerated points (QDPs) with transmission dips (marked with color arrows) maintain. (b) [100] hinge transmission spectra. The hinge transport preserves a high transmission in the surface bandgap region (orange-shaded) in both intact and disordered cases. (c) Response spectra for corner states. The decreased pressure amplitude in the existence of vacancy is attributed to the leakage of energy into neighboring sites. (d) Schematic of the spatial distribution of various disorders on the top facet, lateral hinge, and corner. The field maps for the surface (e-f), hinge (g-h), and corner (i) states are presented, where the disorders are highlighted by white dashed rectangles. In (e) and (g), the line defects are composed of 9- and 4-unit cells with random scale ratios in the *xy* plane: [1.19, 0.86, 1.19, 0.99, 1.12, 0.86, 0.97, 1.17] (from left to right) and [0.91, 1.03, 1.09, 1.06] (from upper to lower), respectively.  Furthermore, we also investigate the transmission spectra and response spectra simulations under various defects, including vacancy, substitution, and line defects. The results are presented in Fig. S17. For surface states, the transmission spectra maintain a high value compared to bulk transmission in the bandgap window, except for dips at the quadratic degenerated point (QDP). It also can be seen that the high-efficiency hinge transmissions still keep. For corner states, the response frequency peaks between with and without vacancy cases only shift less than 10 Hz ($<$0.1%). Thus, once in existence, these multiple boundary states are still robust against various moderate disorders and defects, fundamentally distinct from trivial ones. |

**XI. Note on the losses**

**XI-I. Influence on the transport of sound energy**

| 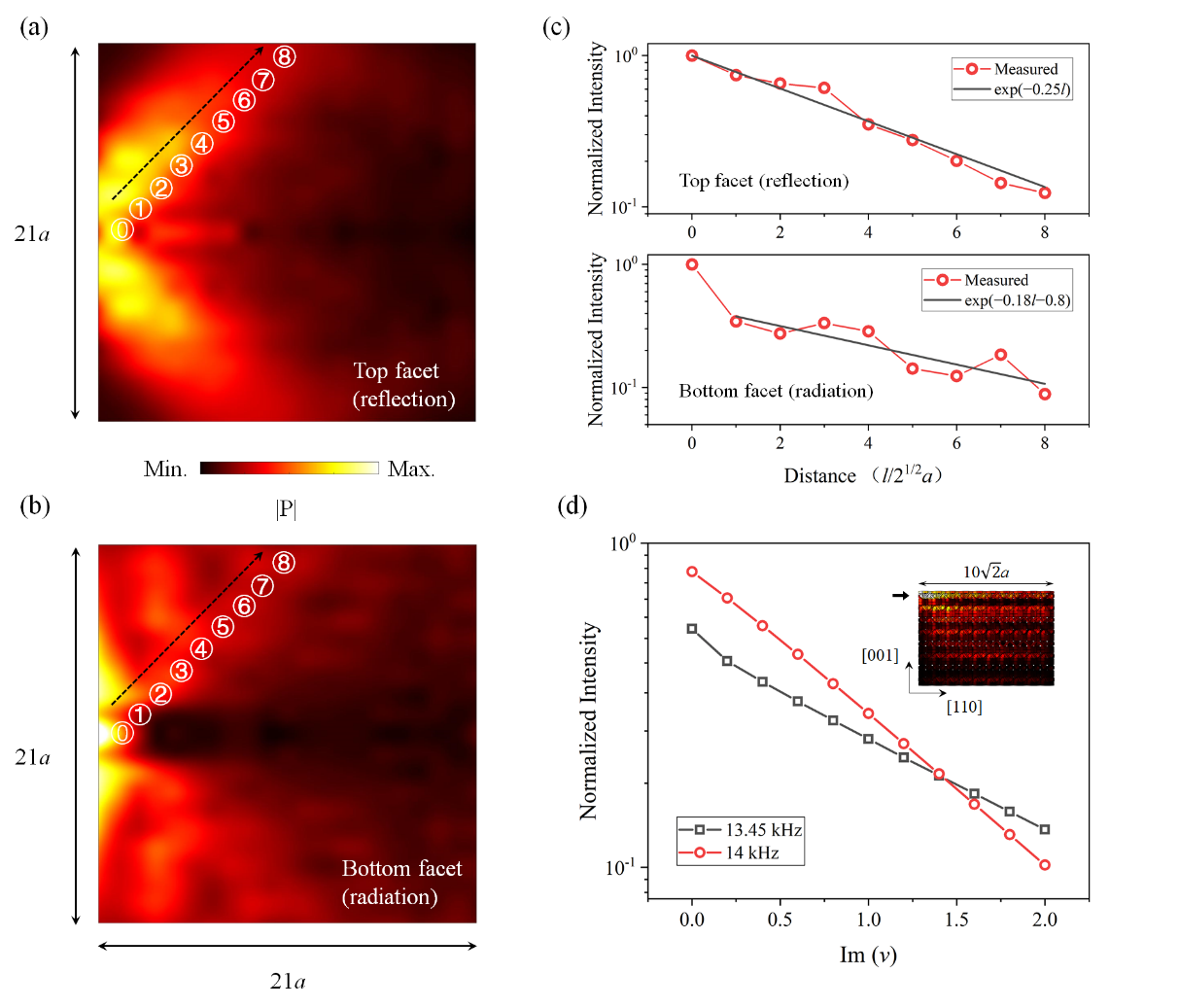 |
| --- |
| **FIG. S18. Influence of losses on the transport of sound energy.** Measured pressure amplitude patterns on the top (a) and bottom (b) facets at 13.45 kHz. (c) Acoustic intensity (red circles) extracted for a sequence of sites along the [110] direction path [black dotted arrows in (a) and (b)]. The black lines show exponential fit, where *l* denotes the propagation length away from site 0. (d) Normalized intensity of reflection top surface state after propagating 10$\sqrt{2}a$ along [110] direction with various losses [imaginary part of sound velocity Im (*v*)] at frequencies 13.45 kHz (black line) and 14 kHz (red line). The inset shows filed distribution (pressure amplitude) at the frequency of 13.45 kHz with Im (*v*)=0. |

In our system, the losses mainly come from two parts. One is absorption (intrinsic loss), and the other is radiation loss (leak to air).

To quantitatively characterize the losses, we experimental scanned the plate-covered top facet and the non-cladding bottom facet in a grid of 21×21 points at the frequency of 13.45 kHz, as shown in Fig. S18(a-b). Then, we extract the sound intensity of measured points along [110] direction (black dashed arrow), corresponding to the $\tilde{\Gamma}\tilde{M}$ direction in surface BZ [Fig. S18(c)].

The transport of sound intensity in the reflection case [upper panel of Fig. S18(c)] follows exponential decay $e^{-0.25l}$ [*l* (in a unit of $\sqrt{2}a$) denotes propagation length], giving a loss coefficient of 0.35 cm^−1^. In the radiation case [lower panel of Fig. S18(c)], the sound energy will rapidly leak into the air surroundings at first (corresponding to the first two unit cells indexed 0 and 1; the energy dissipation coefficient is approximately 0.54 cm^−1^). But, the self-guiding on the radiation surface adjacent to air has a dissipation coefficient of 0.25 cm^−1^ (corresponding to unit cells 1-8). Fig. S18(d) plots the normalized intensity of reflection top surface state after propagating 10$\sqrt{2}a$ along [110] direction with various losses, which are represented by imaginary parts of sound velocity in simulations. The losses are slightly different depending on the operating frequency.

**XI-II. Influence on the response spectra for corner state**

| 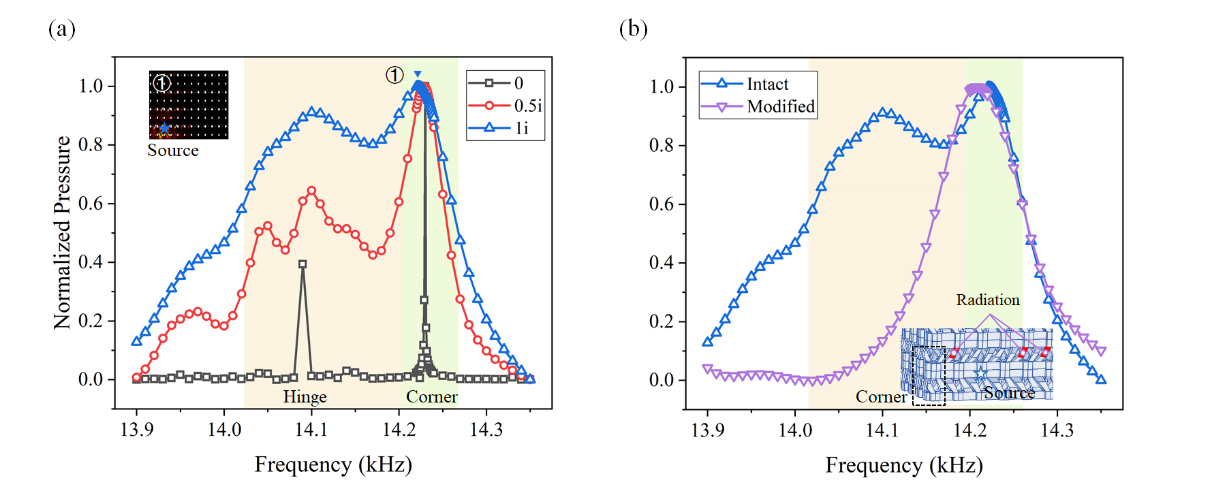 |
| --- |
| **FIG. S19. Influence of losses on response spectra for corner state.** (a) Simulated response spectra under various lossy conditions. As the losses (imaginary part) increase, the original resonance spectra with two sharp peaks for hinge and corner sates (black line) will broaden with low quality factor (blue and red lines). The inset shows field distribution on *xz*-facet with the loss of 1i. (b) Improvement of quality factor via a modified hinge radiation boundary (inset). The imaginary part 1i corresponds to sound velocity (343+1i) *m/s*. |

We examined the influence of losses on the quality factor of response spectra for the corner state. To analyze the loss, we add a nonzero imaginary part to the sound velocity. In the lossless case [black square line in Fig. S19(a)], two sharp peaks correspond to hinge and corner states, respectively. As the loss increases, the widths of response spectra for both corner and hinge states will broaden, then overlap within a narrow hinge bandgap (approximately 60 Hz), finally resulting in a relatively low quality factor. In the case of 1i [blue triangle line, sound velocity (343+1i) *m/s*], the numerical quality factor is approximately 57, which matches our measured result (approximately 53). Therefore, we believe that the experimental data is valid for confirming the existence of the corner state. Furthermore, we show that the quality factor can be improved by adopting a modified radiation hinge configuration to suppress hinge excitation. The improved quality factor is approximately 117 [purple line Fig. S19 (b)]. The quality factor can also be improved by reducing the loss and/or broadening bandgap width, e.g., increasing the contrast ratio of two layers.

In the lossless case, the in-gap corner state is of different frequency from hinge states. They locate on different facets. Thus, they don’t hybridize with each other. But when considering the loss, the co-excitation of both corner and hinge states is possible due to their overlapping response frequencies, as shown in Fig. S19.

On the other hand, our hinge state originates from C*_4z_* symmetry [topological index $\chi^{4}=(-2,-2,1)$], while the corner state originates from *C_2x_* symmetry [topological index $\chi^{2}=(0,0,-1)$]. Such different topological natures may allow us to manipulate them separately. It is possible to construct them at the same frequency, behaving like a bound corner state in the continuum hinge.

**XII. The localization lengths of various boundary modes**

| 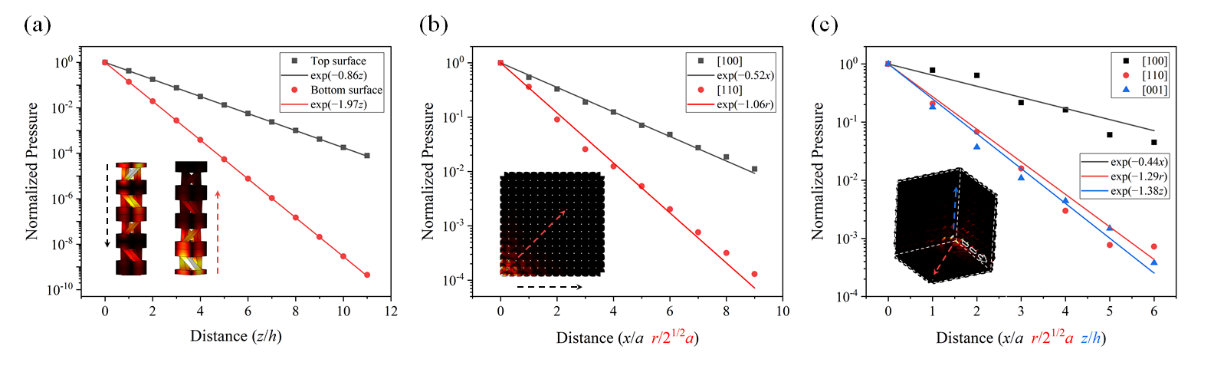 |
| --- |
| **FIG. S20. Estimated localization lengths.** (a) Normalized pressure amplitude for the surface mode at $\tilde{M}$ point extracted within a sequence of unit cells along the *z*-direction [black dots for (001) surface and red dots for (00$\bar{1}$) surface]. The black (red) line shows the exponential fitting result, where *z* denotes the distance from the top (bottom) facet. (b) The spatial dependence of the pressure amplitude for hinge mode with Bloch vector *k*=0.6π/*a* along [100] (black) and [110] (red) directions. The dots and lines are simulated and fitting results, respectively. (c) For the corner mode along three different directions. Insets show field distributions. |

For boundary modes, the decays of pressure amplitudes follow an exponential relation $\left| P \right|\propto e^{{-|r|}/\zeta}$, where |*r*| represents the distance and $\zeta$ represents the localization length. Here, we use four boundary modes (two surface modes on both top and bottom facets, one hinge mode, and one corner mode) at the central frequency of the bandgap to estimate their localization lengths.

Figure S20 shows the normalized pressure amplitude for surface, hinge, and corner states. By fitting the simulated data, we can obtain the corresponding localization lengths: $\zeta_{TS[00\bar{1}]}$=1.16*h,* $\zeta_{BS[001]}$=0.51*h*, $\zeta_{H[100]}$=1.92*a*, $\zeta_{H[110]}$=1.33*a*, $\zeta_{C[100]}$=2.27*a*, $\zeta_{C[110]}$=1.10*a*, and $\zeta_{C[001]}$=0.73*h*, where subscript *TS*, *BS*, *H*, *C* denotes the top surface, bottom surface, hinge modes and corner mode, respectively, and […] specifies directions. Note that the localization lengths increase when the frequencies get close to the band edge.

**Experimental setup**

| 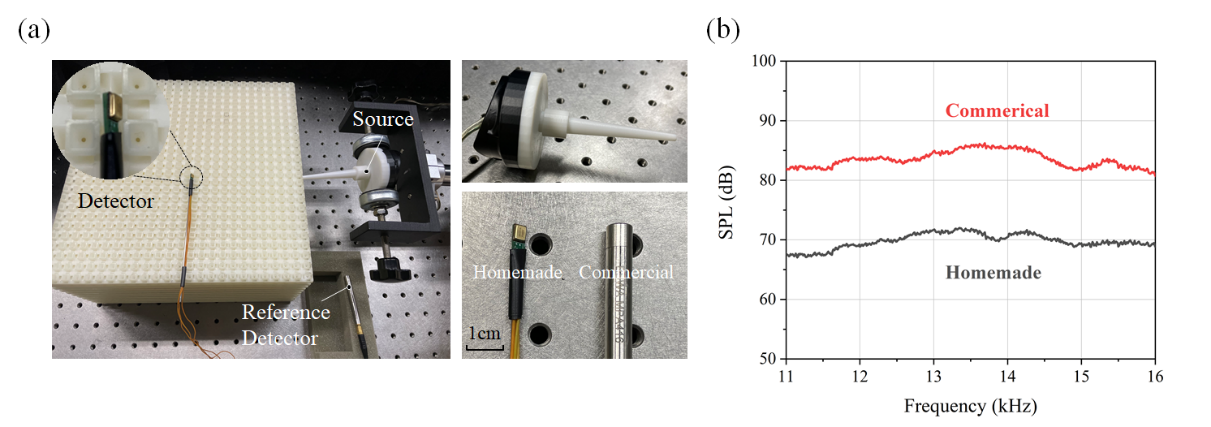 |
| --- |
| **FIG. S21. Details of the experiments.** (a) Pictures of the experimental setup, where a commercial loudspeaker, a homemade microphone, and a commercial microphone are used as the excitation source, measurement detector, and reference detector, respectively. (b) Frequency response of sound pressure level (SPL) for homemade and commercial microphones. |

Our experiments are performed for airborne sounds in an audible frequency window from 13 to 15.1 kHz. In the experiment, a commercial loudspeaker (AMT-47) with a horn acts as a point-like source. The homemade microphone has a full dimension of 5×2×3.5 mm^3^, small enough to insert into the sample to obtain acoustic pressure. Compared to a commercial 1/4-inch microphone (BSWA-MPA416 from BSWA Technology), our homemade microphone shows a similar flat response ($<$5 dB) in the frequency window of 11-16 kHz [Fig. S21(b)]. A little lower acoustic intensity is coming from the smaller size. The detectors are placed 3cm away from the speaker in the test.

Considering the aspect of experimental validity and economical principles, we divide into three small segments from a large crystal to measure the different dimensional boundary states (Fig. S22). Compared to a large-size sample with a full dimension of 25*a*×25*a*×24*h*, the designing strategy using different samples of different sizes (25*a*×25*a*×8*h* for surface, 6*a*×6*a*×24*h* for hinge, and 11*a*×11*a*×5*h* for corner) can effectively save about half of the raw materials. Such division is also convenient for excitation and measurement in experiments under various boundary conditions.

| 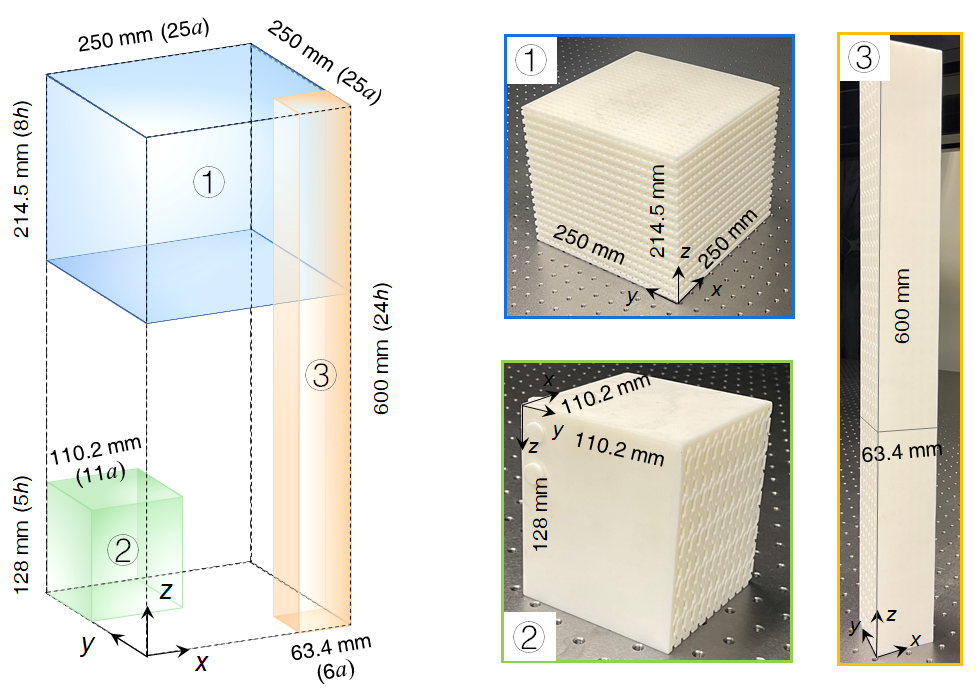 |
| --- |
| **FIG. S22. Schematic of a full size crystal (25*a*×25*a*×24*h*), compared to three small segments.** In experiments, sample 1, 2, and 3 are used to measure surface, corner, and hinge states, respectively. |
| 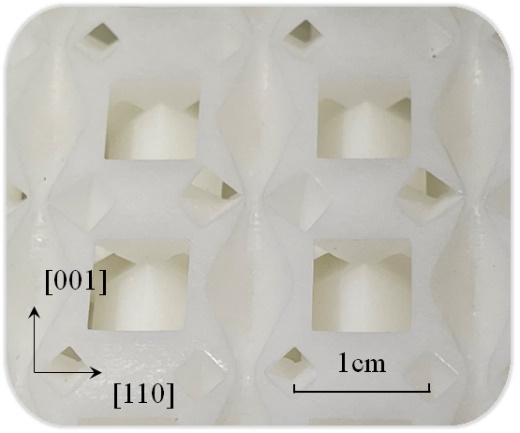 |
| **FIG. S23. Magnified sample details of lateral facet.** |

All samples used in the experiments are fabricated with photosensitive resin (Godart^TM^ 8111X) via 3D printing (tolerance 0.1mm). This stereolithography material (modulus 3160 MPa, density 1.14 g$\cdot$cm^-3^) acts as a hard boundary for sound due to the huge impedance mismatch. The thickness of the cover plate severed as a hard boundary of our samples is 2mm. In the fabrication procedure, we tilt the sample to prevent the use of additional supporting pillars within the structure. As shown in Fig. S23, the sample is well fabricated. The slight surface roughness is acceptable in the experiments due to the robustness of topological states (Fig. S15).
